# Supplementary material for: Succinate uptake by T cells suppresses their effector function via inhibition of mitochondrial glucose oxidation
Source: Cell Rep. 2022 Aug 16;40(7):111193. doi: 10.1016/j.celrep.2022.111193 (PMC9638018; doi:10.1016/j.celrep.2022.111193)
Supplement: Document S1. Figures S1–S4 and Tables S1 and S2 [file mmc1.pdf]

Cell Reports, Volume *40*

Supplemental information

Succinate uptake by T cells suppresses their effector function via inhibition of mitochondrial glucose oxidation

Nancy Gudgeon, Haydn Munford, Emma L. Bishop, James Hill, Taylor Fulton-Ward, David Bending, Jennie Roberts, Daniel A. Tennant, and Sarah Dimeloe

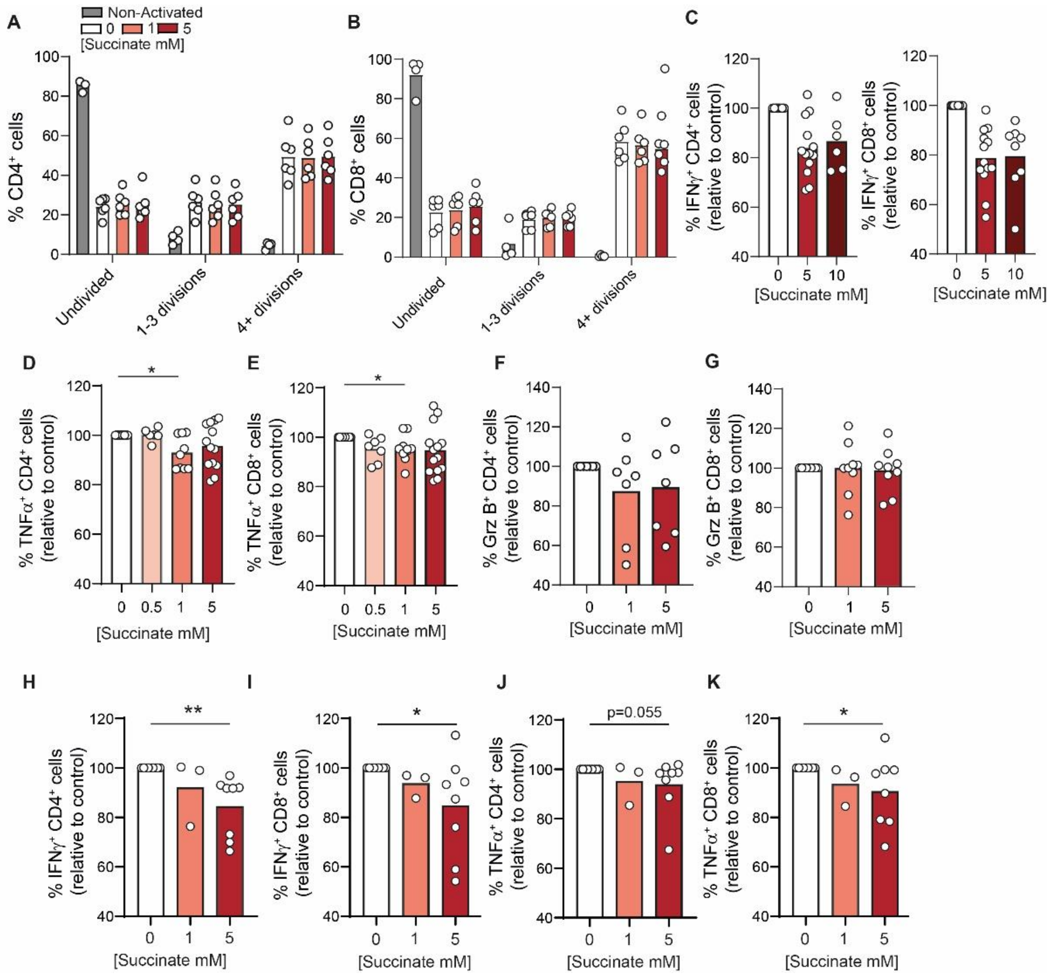

Supplementary Figure 1, related to Figure 1: Effects of succinate on CD4+ and CD8+ T cell proliferation and effector functions.

$CD4^+$ and $CD8^+$ T cells were activated for 72h *in vitro* in presence of exogenous succinate at indicated concentrations, and assessed for **(A-B)** proliferation (dilution of cell-trace violet) (n= 6 independent donors), **(C)** frequency of IFN-γ-expressing cells (n= 6 to 14 independent donors) **(D-E)** frequency of TNF-α-expressing cells (n= 6 to 14 independent donors) and **(F-G)** Granzyme B (GrzB) expressing cells (n= 6 to 9 independent donors) by flow cytometry. **(H-K)** T cells within total human PBMC were activated for 48h in presence of exogenous succinate at indicated concentrations and assessed for frequency of IFN-γ-expressing cells and TNF-α-expressing cells (n= 8 independent donors) by flow cytometry. Bars represent mean data. *p<05, **p<0.01

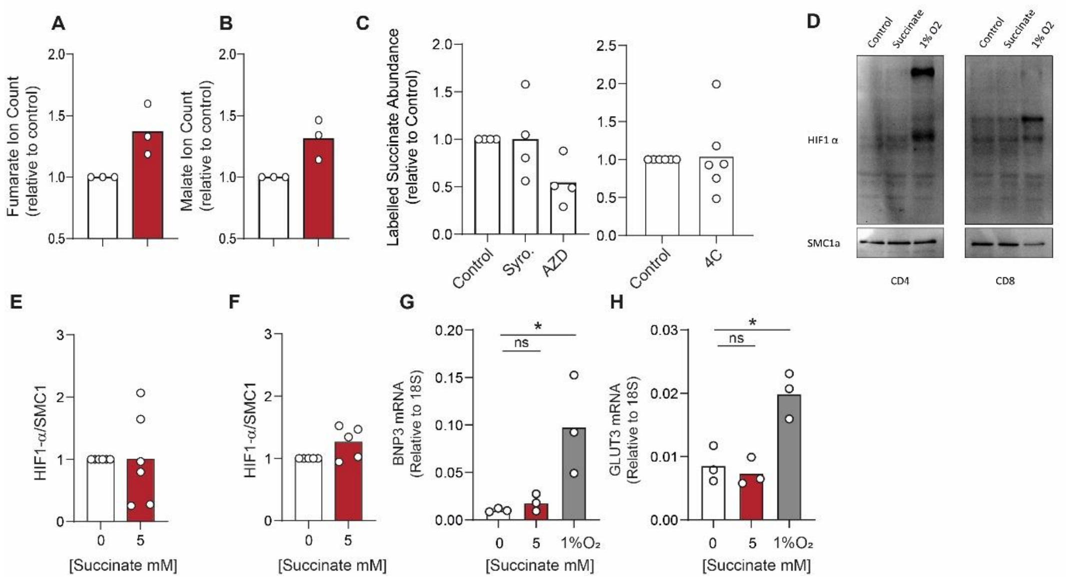

Supplementary Figure 2, related to Figure 2: Effect of succinate on intracellular TCA cycle intermediate abundance and HIF-1α activity in T cells

(A-B) CD4$^+$ T cells were activated for 72h in vitro in presence of 5mM succinate as indicated and assessed for intracellular fumarate and malate abundance (expressed as ion count normalised to the internal standard, D-6-glutaric acid) by GC-MS (n=3 independent donors) **(C)** CD4$^+$ T cells were activated for 72h in presence of 5mM fully ^{13}C-labeled succinate in absence and presence of the MCT1/4 inhibitor syrosingopine (10µM), MCT1 inhibitor AZD-3965 (10µM) or SUCNR1 inhibitor 4C (5µM) and assessed for mass isotopomer distribution (MID) of the succinate by GC-MS (n=3 independent donors). (**D-F**) CD4$^+$and CD8$^+$ T cells were activated for 72h *in vitro* in presence of 5mM succinate and assessed for abundance of HIF-1α by western blot (n=5 independent donors). **(G-H)** CD4$^+$ T cells cultured as in (A) and additionally at 1% atmospheric O_2 were assessed for expression of **(G)** BNIP3 and **(H)** Glut3 by qPCR (n=3 independent donors). Bars represent mean data. *p<05, **p<0.01.

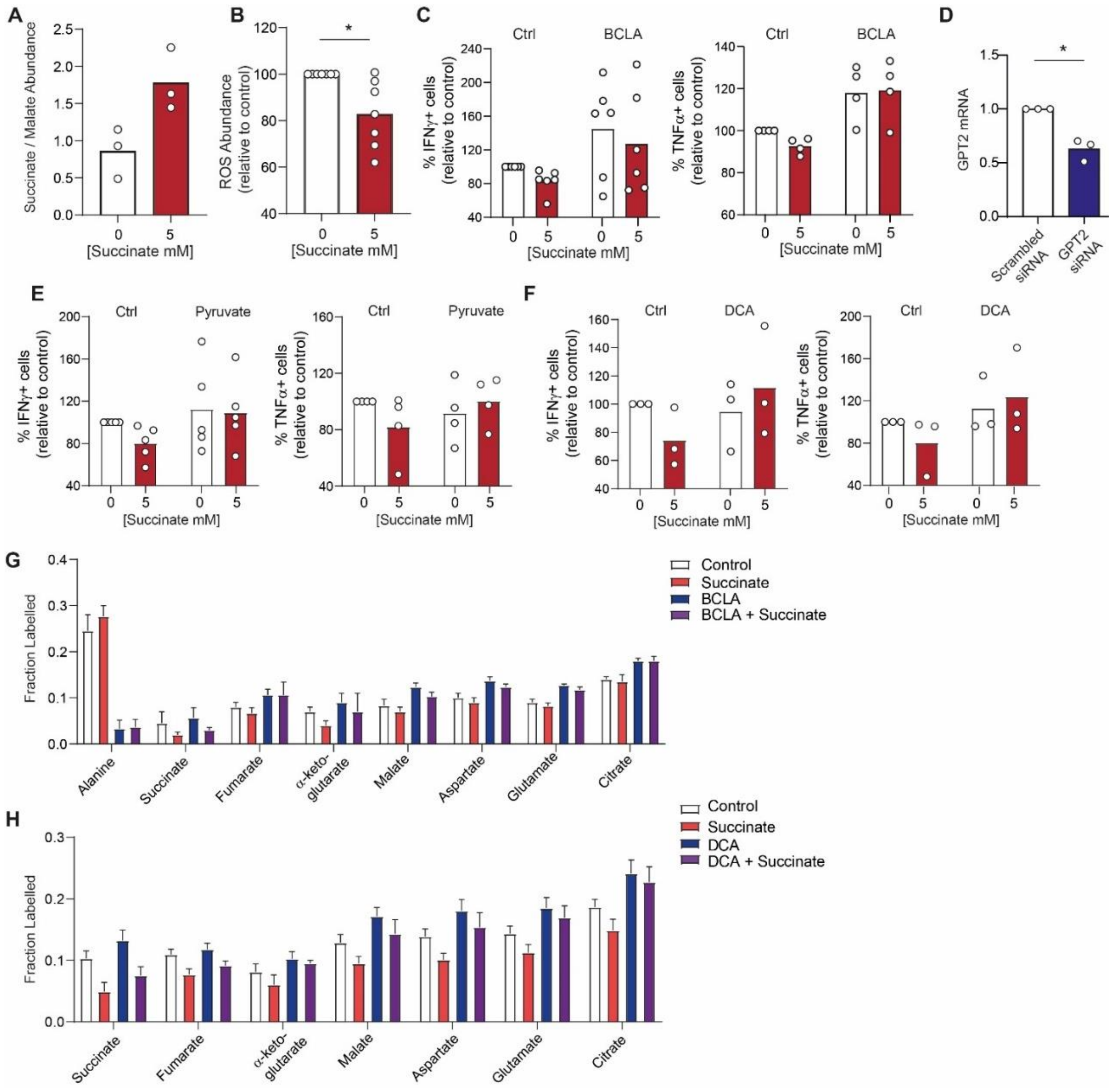

Supplementary Figure 3, related to Figure 3: Succinate impairs T cell mitochondrial activity; interventions to restore mitochondrial glucose oxidation restore T cell effector function

(A) $CD4^+$ T cells were activated for 72h in vitro in presence of 5mM succinate as indicated and assessed for intracellular succinate and malate abundance (expressed as a ratio of the ion counts normalised to the internal standard, D-6-glutaric acid) by GC-MS (n=3 independent donors). (**B**) CD4+ T cells were activated as in (A) and assessed for total ROS abundance by flow cytometry following incubation with 2',7'–dichlorofluorescin diacetate (DCFDA) (n=7 independent donors) **(C)** $CD8^+$ T cells were activated for 72h *in vitro* in presence of exogenous succinate and/or 240µM of the GPT2 inhibitor β-chloro-L-alanine (BCLA) and assessed for assessed for the frequency of IFN-γ- and TNF-α-expressing cells by flow cytometry (n=4 independent donors). **(D)** $CD4^+$ T cells were activated for 72h in vitro in presence of exogenous succinate and/or scrambled or GPT2-targeting siRNA and assessed for GPT2 mRNA abundance by qPCR (n=3 independent donors) **(E-F)** $CD8^+$ T cells were activated as in (D) in presence of succinate and/or pyruvate (10mM) or DCA (10mM) as indicated and assessed for the frequency of IFN-γ- and TNF-α-expressing cells by flow cytometry (n=4/3 independent donors for pyruvate/DCA respectively). Bars represent mean data. **(G-H)** $CD4^+$ T cells were activated for 72h in vitro in presence of 5mM exogenous succinate and/or 240µM β-chloro-L-alanine (BCLA) or 10mM dichloroacetate (DCA) before incubation with fully ^{13}C-labelled glucose assessment of fractional labelling of the indicated metabolites by GC-MS (n=3 independent donors) Mean data +/-SEM are shown for G and H. *p<05, **p<0.01.

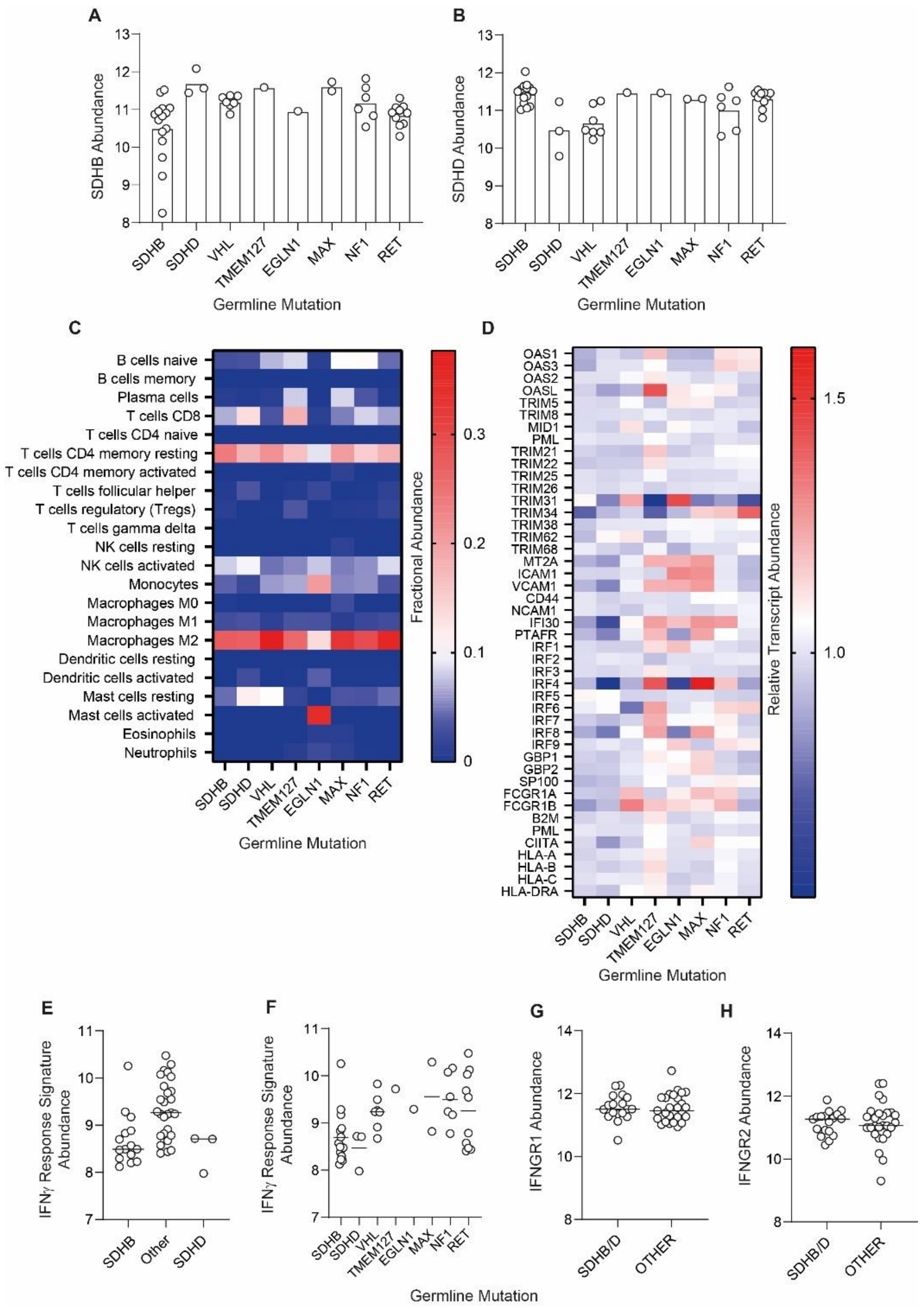

Supplementary Figure 4, related to Figure 4: Gene expression data and immune cell fractional abundance in pheochromocytoma and paraganglioma with germline mutations in SDHB, SDHD or other genes

(A-B) Log2normalised counts for SDHB **(A)** and SDHD **(B)** in tumour samples of pheochromocytoma and paraganglioma with the indicated germline mutations, using RNA-sequencing data generated by Fishbein et al[1]. **(C)** CIBERSORTx analysis of the fractional abundance of indicated immune cell subsets in these samples. **(D)** Abundance of defined IFN-γ response signature transcripts within the same dataset, expressed relative to the mean abundance of each transcript across all samples. **(E-F)** Geometric mean of Log2normalised counts for the IFN-γ response transcripts in these samples, comparing SDHB, SDHD and other germline mutations **(E)** or comparing all germline mutations separately **(F)**. **(G-H)** Log2normalised counts for IFNGR1 **(G)** and IFNGR2 **(H)** in samples with the indicated germline mutations Lines represent mean data.

Supplementary Table 1, related to STAR methods: Details of primers used for SYBRgreen qPCR analysis.

**Gene**	**Forward**	**Reverse**
BNIP3	CAGGGCTCCTGGGTAGAACT	CTACTCCGTCCAGACTCATGC
GLUT3	GCTGGGCATCGTTGTTGGA	GCACTTTGTAGGATAGCAGGAAG
GPT2	GACCCCGACAACATCTACCTG	TCATCACACCTGTCCGTGACT

Supplementary Table 2, related to Figure 4 and supplementary Figure 4: Details of IFN-γ signature genes in Fig. 4/Supp. Fig. 4, constructed using Reactome pathway browser

Gene	Protein
OAS1	2'-5'-oligoadenylate synthetase 1
OAS3	2'-5'-oligoadenylate synthetase 3
OAS2	2'-5'-oligoadenylate synthetase 2
OASL	2'-5'-oligoadenylate synthase-like protein
TRIM5	Tripartite motif-containing protein 5
TRIM8	Tripartite motif-containing protein 8
MID	MID1 / Tripartite motif-containing protein 18
PML	PML Nuclear Body Scaffold/ Tripartite motif-containing protein 19
TRIM21	Tripartite motif-containing protein 21
TRIM22	Tripartite motif-containing protein 22
TRIM25	Tripartite motif-containing protein 23
TRIM26	Tripartite motif-containing protein 26
TRIM31	Tripartite motif-containing protein 31
TRIM34	Tripartite motif-containing protein 34
TRIM38	Tripartite motif-containing protein 38
TRIM62	Tripartite motif-containing protein 62
TRIM68	Tripartite motif-containing protein 68
MT2A	Metallothienin 2A
ICAM1	Intercellular Adhesion Molecule 1
VCAM1	Vascular Cell Adhesion Molecule 1
CD44	CD44 / HCAM (homing cell adhesion molecule)
NCAM1	Neural Cell Adhesion Molecule 1
IFI30	Gamma-interferon-inducible lysosomal thiol reductase
PTAFR	Platelet-activating factor receptor
IRF1	Interferon regulatory factor 1
IRF2	Interferon regulatory factor 2
IRF3	Interferon regulatory factor 3
IRF4	Interferon regulatory factor 4
IRF5	Interferon regulatory factor 5
IRF6	Interferon regulatory factor 6
IRF7	Interferon regulatory factor 7
IRF8	Interferon regulatory factor 8
IRF9	Interferon regulatory factor 9
GBP1	Guanylate-binding protein 1
GBP2	Guanylate-binding protein 2
SP100	Nuclear autoantigen Sp-100
FCGR1A	High affinity immunoglobulin gamma Fc receptor I
FCGR1B	High affinity immunoglobulin gamma Fc receptor IB
B2M	Beta 2 microglobulin
PML	Protein PML
CIITA	MHC class II transactivator
HLA-A	HLA-A
HLA-B	HLA-B
HLA-C	HLA-C
HLA-DRA	HLA-DR
