# Supplementary material for: Succinate uptake by T cells suppresses their effector function via inhibition of mitochondrial glucose oxidation
Source: Cell Rep. 2022 Aug 16;40(7):111193. doi: 10.1016/j.celrep.2022.111193 (PMC9638018; doi:10.1016/j.celrep.2022.111193)
Supplement: Document S2. Article plus supplemental information [file mmc2.pdf]

Cell Reports

Report

Succinate uptake by T cells suppresses their effector function via inhibition of mitochondrial glucose oxidation

Graphical abstract

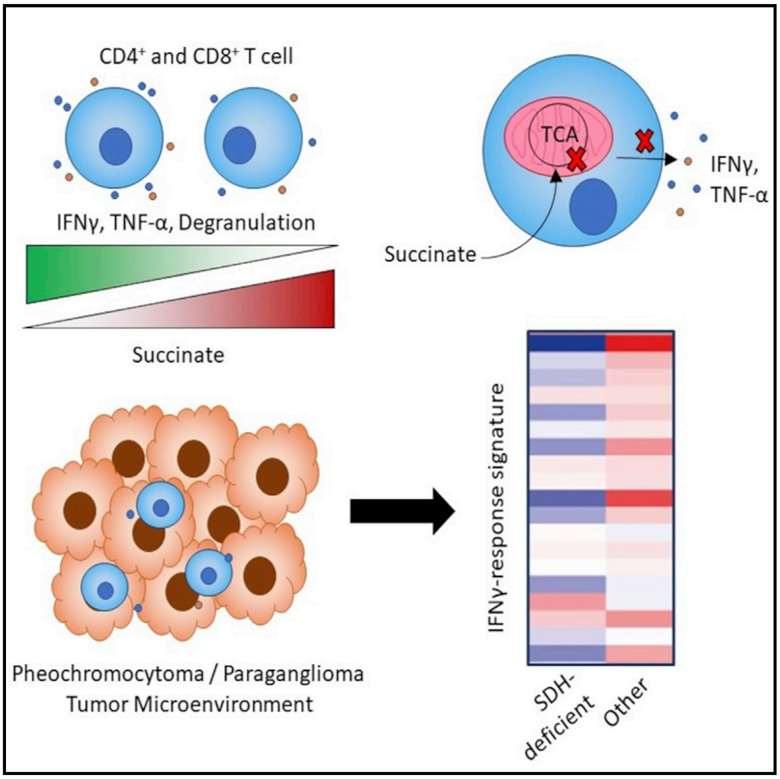

Highlights

- Succinate inhibits $CD4^+$ and $CD8^+$ T cell effector function, including IFN-γ secretion
- T cells readily assimilate succinate from their environment
- Succinate inhibits T cell succinyl-CoA synthetase activity and the TCA cycle
- Neuroendocrine tumors with succinate dehydrogenase mutations have poor IFN-γ responses

Authors

Nancy Gudgeon, Haydn Munford, Emma L. Bishop, ..., Jennie Roberts, Daniel A. Tennant, Sarah Dimeloe

Correspondence

s.k.dimeloe@bham.ac.uk

In brief

Succinate dehydrogenase (SDH) mutations drive succinate accumulation in tumor microenvironments. Gudgeon et al. observe that succinate suppresses T cell degranulation and cytokine secretion, particularly IFN-γ. This is associated with succinate uptake, inhibited succinyl-CoA synthetase activity, and impaired tricarboxylic acid cycle flux. Consistently, IFN-γ-induced genes are suppressed in SDH-deficient neuroendocrine tumors.

Gudgeon et al., 2022, Cell Reports *40*, 111193
August 16, 2022 © 2022 The Author(s).
https://doi.org/10.1016/j.celrep.2022.111193

CellPress

Cell Reports

CellPress
OPEN ACCESS

Report

Succinate uptake by T cells suppresses their effector function via inhibition of mitochondrial glucose oxidation

Nancy Gudgeon,[1,3] Haydn Munford,[1,3] Emma L. Bishop,[1] James Hill,[1] Taylor Fulton-Ward,[1,2] David Bending,[1] Jennie Roberts,[2] Daniel A. Tennant,[2] and Sarah Dimeloe[1,2,4,*]

[1]Institute of Immunology and Immunotherapy, College of Medical and Dental Sciences, University of Birmingham, Birmingham, UK

[2]Institute of Metabolism and Systems Research, College of Medical and Dental Sciences, University of Birmingham, Birmingham, UK

[3]These authors contributed equally

[4]Lead contact

*Correspondence: s.k.dimeloe@bham.ac.uk

https://doi.org/10.1016/j.celrep.2022.111193

SUMMARY

Succinate dehydrogenase (SDH) loss-of-function mutations drive succinate accumulation in tumor microenvironments, for example in the neuroendocrine tumors pheochromocytoma (PC) and paraganglioma (PG). Control of innate immune cell activity by succinate is described, but effects on T cells have not been interrogated. Here we report that exposure of human $CD4^+$ and $CD8^+$ T cells to tumor-associated succinate concentrations suppresses degranulation and cytokine secretion, including of the key anti-tumor cytokine interferon-γ (IFN-γ). Mechanistically, this is associated with succinate uptake—partly via the monocarboxylate transporter 1 (MCT1)—inhibition of succinyl coenzyme A synthetase activity and impaired glucose flux through the tricarboxylic acid cycle. Consistently, pharmacological and genetic interventions restoring glucose oxidation rescue T cell function. Tumor RNA-sequencing data from patients with PC and PG reveal profound suppression of IFN-γ-induced genes in SDH-deficient tumors compared with those with other mutations, supporting a role for succinate in modulating the anti-tumor immune response *in vivo*.

INTRODUCTION

Oncometabolites are small-molecule metabolic constituents that accumulate in tumor microenvironments and initiate or promote tumor growth. This activity is substantially exerted on tumor cells (Sciacovelli and Frezza, 2016), but it is increasingly apparent that infiltrating immune cells are also affected. For example, lactate, abundant in many tumors, impairs T cell function via effects on cellular metabolism, redox status, and transcription factor function (Quinn et al., 2020; Ratter et al., 2018), while 2-hydroxyglutarate (2-HG), which accumulates in context of isocitrate dehydrogenase gain-of-function mutations, suppresses T cell calcium signaling and transcriptional activity (Bunse et al., 2018). The tricarboxylic acid (TCA) cycle intermediate succinate reaches high levels in tumors with loss-of-function mutations in succinate dehydrogenase (SDH) subunits B and D (SDHB and SDHD). These occur in the rare neuroendocrine tumors pheochromocytoma (PC) and paraganglioma (PG), originating from chromaffin cells in the adrenal medulla (PC) or sympathetic/parasympathetic ganglia (PG). These tumors are mostly benign but up to 25% are malignant, metastasizing to non-chromaffin tissues and associated with poor survival. SDHB germline mutations are a risk factor for this and are associated with diminished expression of chromaffin differentiation genes. This has been linked to succinate-mediated inhibition of α-ketoglutarate-dependent dioxygenases (α-KGDDs) including histone/DNA demethylases (Letouze et al., 2013) and prolyl hydroxylase (PHD), causing hypoxia-inducible factor 1α (HIF-1α) stabilization (Selak et al., 2005).

Both pro- and anti-inflammatory effects of succinate on innate immune cells have been identified (Harber et al., 2020; Mills et al., 2016; Tannahill et al., 2013) and have been described to influence intestinal immune homeostasis (Lei et al., 2018; Rubic et al., 2008), obesity (Keiran et al., 2019), chronic neuroinflammation (Peruzzotti-Jametti et al., 2018), systemic lupus erythematosus (Caielli et al., 2019), and lung cancer (Wu et al., 2019). These effects were linked to recognition via the G-protein-coupled receptor SUCNR1 as well as intracellular activity. For example, succinate is chemotactic for dendritic cells and enhances their secretion of pro-inflammatory cytokines via SUCNR1 (Rubic et al., 2008), which elicits T helper 17 responses in experimental arthritis (Saraiva et al., 2018). Similarly, intestinal tuft cell SUCNR1 mediates induction of type 2 immune responses by dietary succinate and altered microbiome components (Nadjsombati et al., 2018)(Lei et al., 2018). In macrophages, succinate was found to promote inflammatory interleukin-1β (IL-1β) expression while suppressing expression of anti-inflammatory IL-10 and IL-1Ra. This was attributed to intracellular effects of succinate, specifically PHD inhibition (Tannahill et al., 2013) and generation of reactive oxygen species (ROS) through SDH-meditated

© 2022 The Author(s).
This is an open access article under the CC BY license (http://creativecommons.org/licenses/by/4.0/).

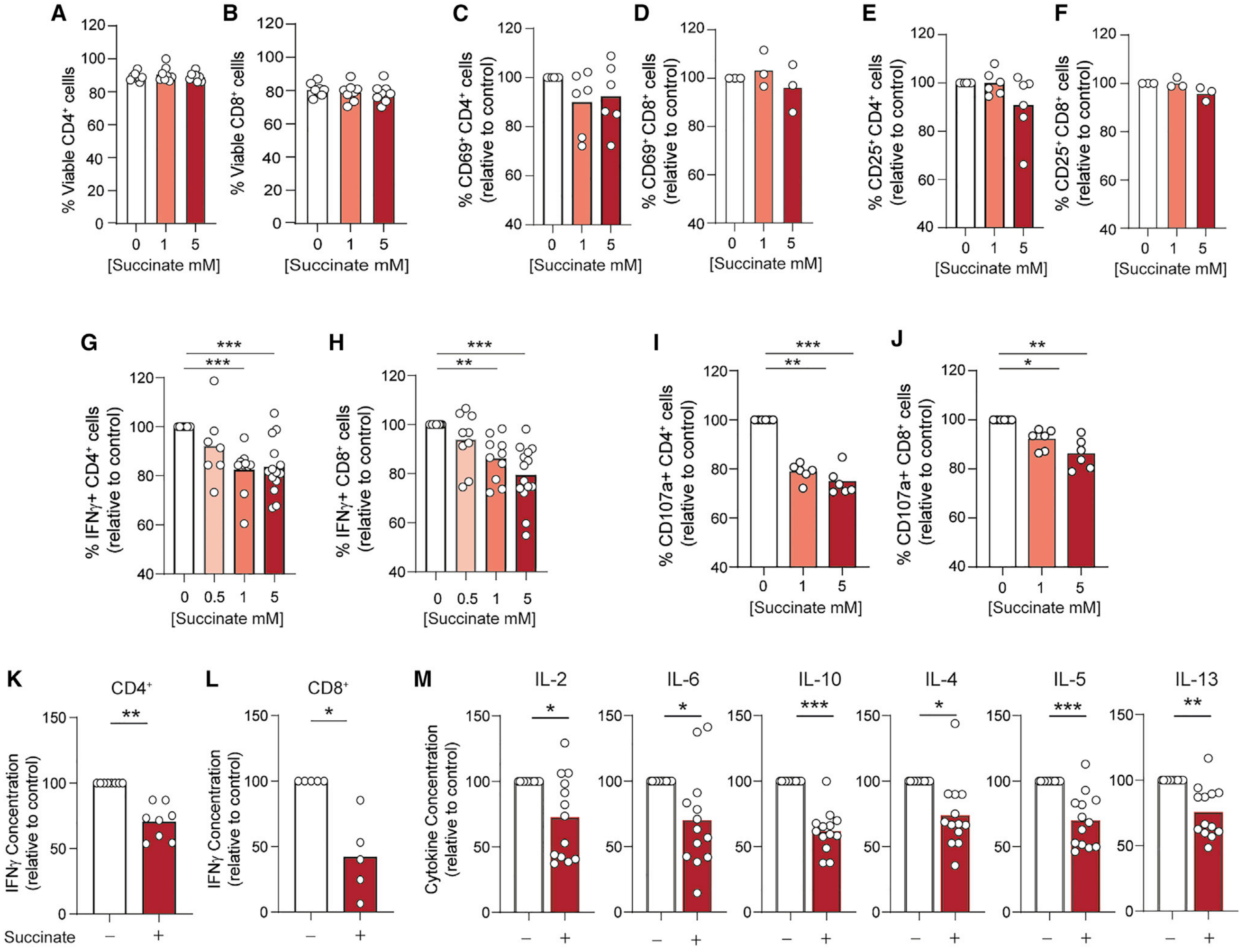

Figure 1. T cell cytokine expression and degranulation is suppressed by tumor-associated succinate levels

(A–J) Human CD4+ (A, C, E, G, and I) or CD8+ (B, D, F, H, and J) T cells were activated for 72 h in the presence of indicated succinate concentrations and assessed for (A and B) viability (n = 8 independent donors), frequency of (C–F) CD25- and CD69-expressing cells (n = 6 and n = 3 independent donors for CD4+ and CD8+ T cells, respectively) (G and H) IFN-γ-expressing cells (n = 6–14 independent donors), and (I and J) CD107a externalization (n = 6 independent donors) by flow cytometry.

(K–M) Supernatants from cells activated as in (A) to (J) were assessed for (K and L) IFN-γ by ELISA (n = 8 and n = 5 independent donors for CD4+ and CD8+ T cells, respectively) or (M) indicated cytokine by multiplex cytokine bead array (n = 13 independent donors). ELISA and multiplex cytokine data are corrected for cell number.

Bars represent mean data. *$p < 0.05$, **$p < 0.01$, ***$p < 0.005$.

succinate oxidation (Mills et al., 2016). Conversely, recent studies also indicate anti-inflammatory roles for succinate in macrophages. Specifically, succinate treatment of adipose-tissue-derived macrophages decreased inflammatory cytokine expression (Keiran et al., 2019), which agrees with findings in an experimental lung cancer model where succinate treatment promoted cancer progression via macrophage polarization toward tumor-permissive phenotypes (Wu et al., 2019).

To date, effects of succinate on adaptive immune cells have not been interrogated in depth. We therefore set out to investigate the effects of elevated succinate levels on T cell immune function and the implications of this in SDH-deficient malignancies.

RESULTS

To explore whether pathological succinate abundance (up to 8–9 mM in PG tumors [Matlac et al., 2021; Richter et al., 2014]) impacts T cell function, we activated human CD4+ and CD8+ T cells under increasing concentrations *in vitro*. T cell viability (Figures 1A and 1B), activation (Figures 1C–1F) and proliferation (Figures S1A and S1B) were unchanged by succinate exposure, but frequencies of CD4+ and CD8+ T cells expressing interferon-γ (IFN-γ) were significantly reduced. Increasing IFN-γ suppression occurred between 0.5 and 10 mM succinate and was maximal at 5 mM and above (Figures 1G, 1H, and S1C). CD4+ and CD8+ T cell degranulation (externalization of

lysosomal-associated membrane protein 1 [LAMP-1/CD107a]) was also impaired by succinate exposure (Figures 1I and 1J). Consistent with fewer IFN-γ-expressing cells, total IFN-γ secreted by $CD4^+$ and $CD8^+$ T cells was also decreased, by 40% and 60%, respectively (Figures 1K and 1L). Frequencies of tumor necrosis factor α (TNF-α)-expressing $CD4^+$ and $CD8^+$ T cells were also reduced by succinate, albeit less than IFN-γ (Figures S1D and S1E). Expression of granzyme B was not altered by succinate exposure (Figures S1F and S1G). To extend analyses of cytokine expression by $CD4^+$ T cells, we performed a multiplex assay. This identified that IL-2, IL-6, IL-10, IL-4, IL-5, and IL-13 were reduced similarly to IFN-γ (Figure 1M), revealing broad effects of succinate on $CD4^+$ T cell cytokine secretion. Succinate can both promote and suppress innate immune cell activity, which may indirectly impact T cell function, meaning observed effects in isolated T cells do not occur in complex cell systems. To check this we assessed cytokine expression by T cells within total peripheral blood mononuclear cells. Here, fewer IFN-γ- and TNF-α-expressing $CD4^+$ and $CD8^+$ T cells were also observed upon succinate exposure (Figures S1H–S1K), indicating overall suppression of T cell function even in the presence of innate immune cells.

SUCNR1 mediates certain effects of succinate in innate immune cells. We therefore interrogated its expression in T cells to understand whether it may mediate the suppression observed. This identified basal SUCNR1 expression in quiescent $CD4^+$ and $CD8^+$ T cells, and further that it is downregulated upon activation, with protein and mRNA levels substantially reduced by 48–72 h irrespective of succinate exposure (Figures 2A–2D). To directly probe whether SUCNR1 mediates the inhibitory effects of succinate in T cells we used the specific antagonist, 4C. 4C treatment alone slightly decreased the frequency of IFN-γ-expressing $CD8^+$ but not $CD4^+$ T cells. Additionally, IFN-γ suppression by succinate was partly blunted by 4C, indicating that signaling through SUCNR1 partially explains T cell suppression by succinate (Figure 2E). Therefore, to further understand how succinate suppresses T cell function, we investigated additional mechanisms. For example, elevated intracellular succinate levels alter cell function via inhibition of α-KGDDs. It remains unclear whether T cells take up succinate from their environment. We therefore first tested this by gas chromatography-mass spectrometry (GC-MS) analysis of $CD4^+$ T cell lysates. This identified that cells activated in the presence of 5 mM succinate had on average 3-fold higher intracellular succinate abundance than those under control conditions (Figure 2F). To further confirm that T cells take up exogenous succinate, $CD4^+$ T cells were activated in the presence of 5 mM ^{13}C-labeled sodium succinate and analyzed by GC-MS for incorporation of the ^{13}C label into the intracellular metabolite pool. This confirmed that most of the intracellular succinate pool was derived from extracellular [^{13}C] succinate, consistent with the large increase in overall abundance (Figure 2G). Additionally, it identified that extracellular succinate was partly metabolized by $CD4^+$ T cells, since the ^{13}C label was detected to a lesser extent in fumarate, malate, citrate, aspartate, and glutamate (Figure 2G). The abundance of fumarate and malate was also increased by 1.5-fold (Figures S2A and S2B). It was recently identified that the monocarboxylate transporter, MCT1, transports succinate in its protonated form (Reddy et al., 2020), which is present under acidic conditions similar to the microenvironment of activated, glycolytic T cells. To probe a role for MCT1 mediating T cell succinate uptake, $CD4^+$ T cells were activated in the presence of [^{13}C]succinate together with the MCT1 inhibitor, AZD-3965. Abundance of ^{13}C-labeled succinate, fumarate, malate, aspartate, citrate, and glutamate was decreased by 30%–40% upon AZD-3965 treatment (Figure 2H), indicating that MCT1 partly mediates succinate uptake in T cells. Conversely, treatment with syrosingopine, a dual MCT1/4 inhibitor with 60-fold higher MCT4 potency, did not alter succinate uptake; thus, MCT4 likely does not play a role (Figure S2C). Similarly, treatment with 4C did not alter [^{13}C] succinate labeling (Figure S2C), indicating that SUCNR1 does not regulate $CD4^+$T cell succinate uptake as is described in macrophages (Peruzzotti-Jametti et al., 2018).

Having confirmed that extracellular succinate is taken up by $CD4^+$ T cells, the impact on HIF-1α abundance and activity was assessed. HIF-1α is reported as both a positive and negative regulator of T cell activity (Finlay et al., 2012; Thiel et al., 2007); therefore, it was pertinent to interrogate for control of this by succinate. Analysis, however, indicated no consistent change in HIF-1α protein abundance in $CD4^+$ or $CD8^+$ T cells activated in the presence of succinate (Figures S2D–S2F). Moreover, mRNA abundance of two HIF-1α target genes, BNIP3 and GLUT3, was not changed in $CD4^+$ T cells exposed to succinate, despite induction in cells activated under hypoxia (Figures S2G and S2H). Taken together, these data indicate that stabilization of HIF-1α is unlikely to mediate the observed suppressive effects of succinate on T cells.

Immune function of T cells is tightly linked to their metabolic activity (Bantug et al., 2018b). The substantial incorporation of succinate into the intracellular pool (Figure 2G) raised the possibility that oxidation of this additional metabolic substrate may augment the overall mitochondrial activity of T cells. To test for this, mitochondrial membrane potential of $CD4^+$ and $CD8^+$ T cells was measured with MitoSpy Orange, a membrane-potential-sensitive fluorescent mitochondrial probe. This identified diminished rather than increased mitochondrial membrane potential in T cells exposed to succinate (Figures 3A and 3B), indicating decreased rather than increased mitochondrial oxidative metabolism. To confirm this we performed extracellular flux analysis of $CD4^+$ T cells (Figures 3C–3J), which identified consistently decreased ATP-coupled oxygen consumption rates (OCR) in $CD4^+$ T cells activated in the presence of 5 mM succinate (Figures 3C and 3E) alongside no change in glycolytic extracellular acidification rate (ECAR) (Figures 3D and 3F). Consistently, ratios of OCR to ECAR were also decreased (Figure 3G). Finally, calculation of ATP production rates from these data identified decreased mitochondrial and overall ATP production alongside unchanged rates of glycolytic ATP production (Figures 3H–3J). Taken together, these data indicate that exposure to succinate impairs $CD4^+$ T cell mitochondrial oxidative function, which was further indicated by elevated succinate/malate ratios in succinate-exposed cells (Figure S3A) and decreased generation of ROS (Figure S3B). Consequently, mitochondrial and overall ATP production is significantly impaired. To probe this metabolic impairment in more detail, we next performed GC-MS analysis to trace the fate of ^{13}C-labeled glucose in $CD4^+$ T cells previously

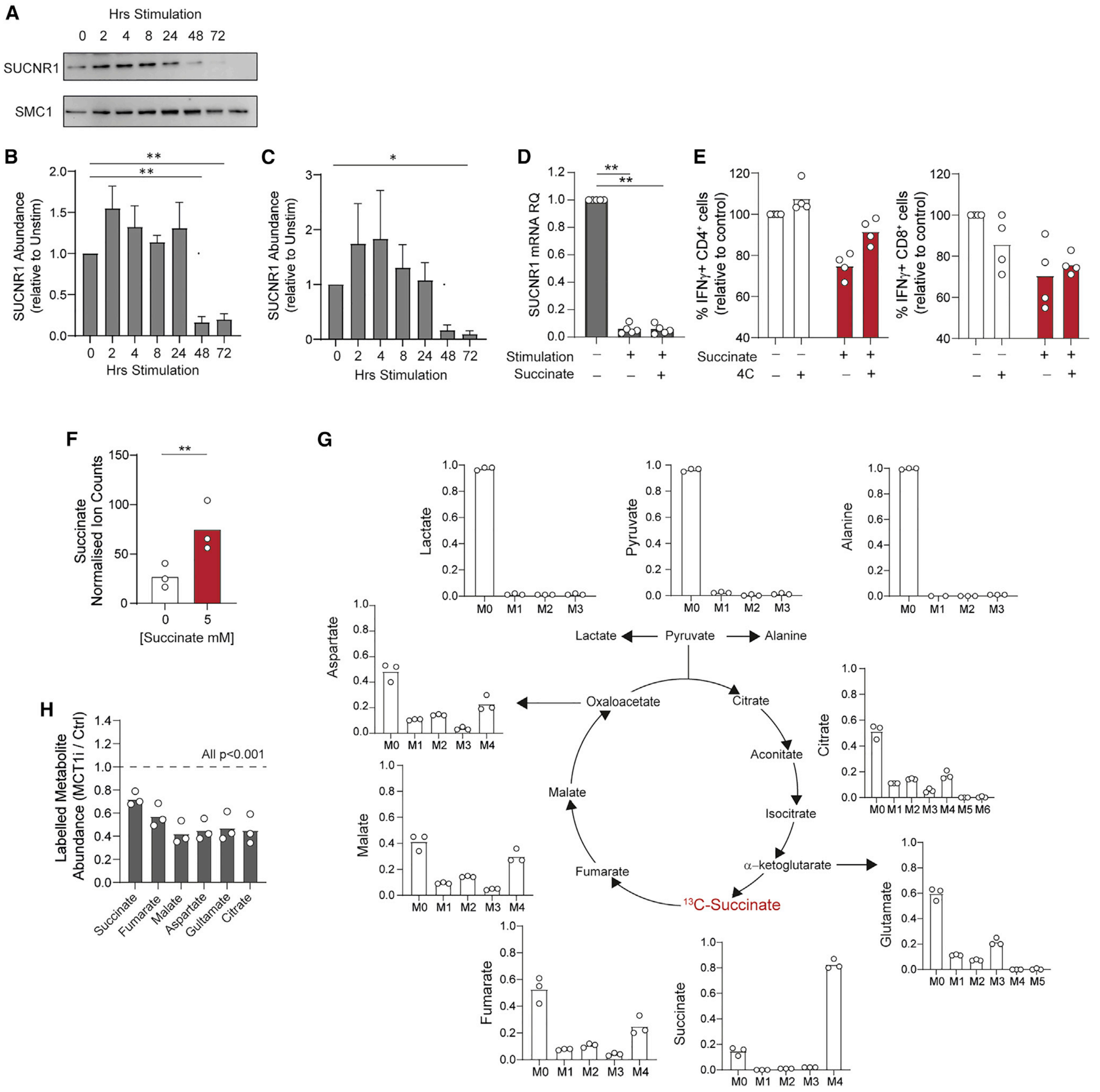

Figure 2. T cells downregulate SUCNR1 upon activation and take up extracellular succinate partly via MCT1

(A–D) CD4+ (A, B, and D) and CD8+ (C) T cells were activated for the indicated time in the presence of 5 mM succinate where indicated and assessed for abundance of SUCNR1 (A–C) protein by western blot (n = 4 independent donors) and (D) mRNA by qPCR (n = 5 independent donors).

(E) CD4+ and CD8+ T cells were activated for 72 h in the presence of exogenous succinate and/or the SUCNR1 antagonist 4C (5 μM) and assessed for frequency of IFN-γ-expressing cells by flow cytometry (n = 4 independent donors).

(F) CD4+ T cells were activated for 72 h in the presence of 5 mM succinate as indicated and assessed for intracellular succinate abundance (expressed as ion count normalized to the internal standard, D-6-glutaric acid) by GC-MS (n = 3 independent donors).

(G and H) CD4+ T cells were activated for 72 h in the presence of 5 mM fully ^{13}C-labeled succinate and assessed for (G) mass isotopomer distribution (MID) of indicated metabolites and (H) abundance of indicated ^{13}C-labeled metabolites in the absence and presence of the MCT1 inhibitor, AZD-3965 (10 μM), by GC-MS (n = 3 independent donors).

Bars represent mean data. Error bars (B and C) represent SEM. *p < 0.05, **p < 0.01, ***p < 0.005.

CellPress
OPEN ACCESS

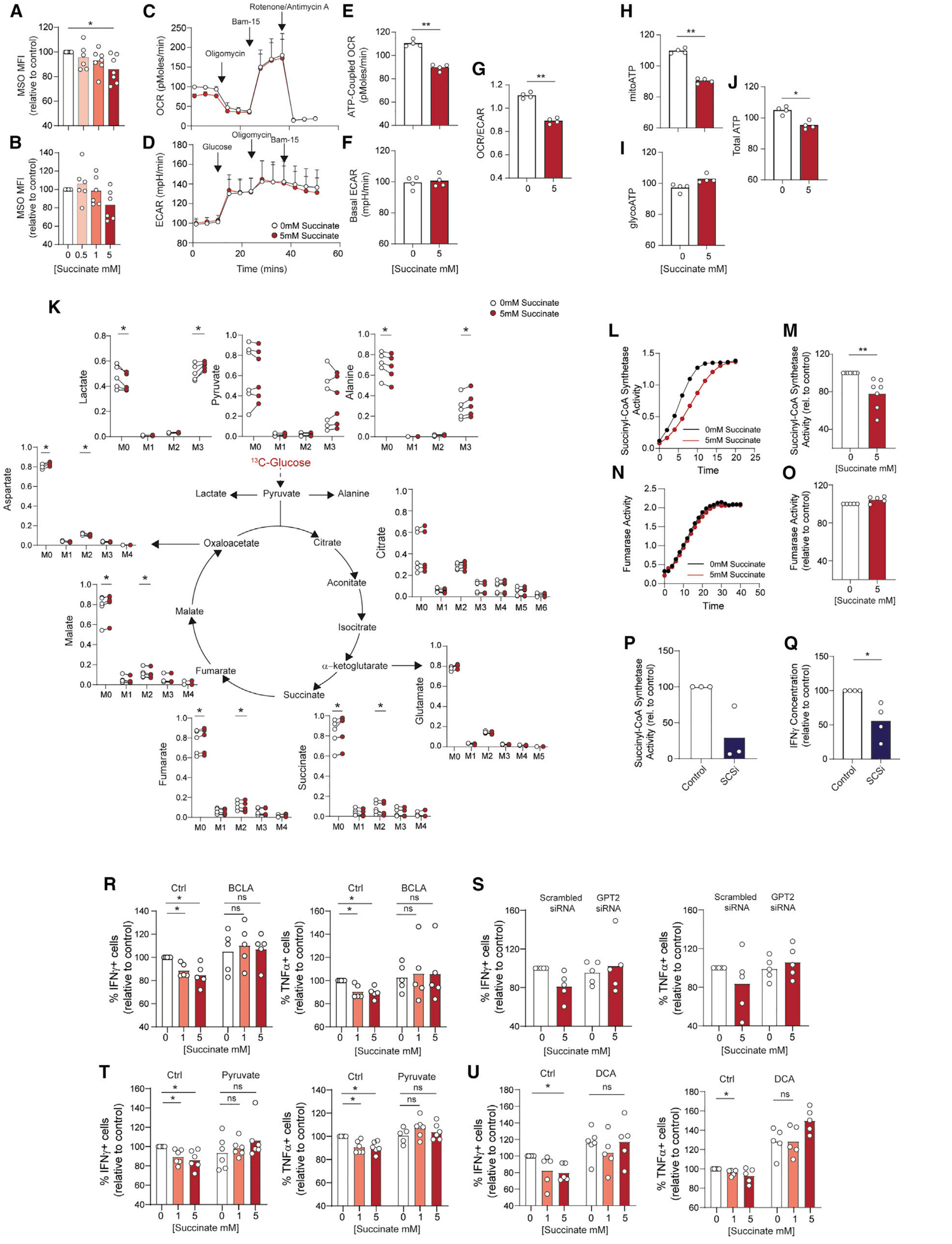

(legend on next page)

activated under control or elevated succinate conditions. Succinate was not present during the glucose labeling period. This identified that following succinate exposure, ^{13}C labeling was consistently increased in both lactate and alanine, alternative products of pyruvate when it is not converted to acetyl coenzyme A (CoA) for oxidation in the TCA cycle (Figure 3K). Moreover, incorporation of glucose into succinate, malate, glutamate, and aspartate was significantly decreased. Taken together, these observations indicate that $CD4^+$ T cell succinate uptake diminishes flux of glucose-derived pyruvate through the TCA cycle. An explanation for this could be that succinate buildup inhibits the upstream TCA cycle enzyme, succinyl-CoA synthetase (SCS), by product inhibition, as is proposed in a kinetic model (Li et al., 2013). To directly test this, SCS activity was biochemically measured in $CD4^+$ T cells activated under control conditions or in the presence of 5 mM succinate. These assays clearly identified diminished SCS activity in succinate-exposed cells (Figures 3L and 3M) while parallel assays of fumarase activity identified no effect of succinate exposure, indicating a specific effect on SCS (Figures 3N and 3O). Importantly, treatment with an established SCS inhibitor (sodium metavanadate [Křivánek and Nováková, 1991], SCSi) inhibited SCS in $CD4^+$ T cells (Figure 3P) and was sufficient to reduce IFN-γ secretion (Figure 3Q), indicating that efficient SCS activity supports T cell effector function.

Next, to further confirm this mechanism, we explored whether impaired T cell function in the presence of succinate could be rescued by correcting this blockade. To this end, we undertook complementary approaches to restore pyruvate flux through the TCA cycle. First, the enzyme glutamic-pyruvic transaminase 2 (GPT2) was targeted. This catalyzes the reversible transamination converting pyruvate into alanine and is inhibited by β-chloro-L-alanine (BCLA). Analysis of cytokine expression identified that BCLA did not alter frequencies of IFN-γ or TNF-α $CD4^+$ or $CD8^+$ T cells expressing cells per se but did restore succinate-mediated inhibition of these cytokines (Figures 3R and S3C). As an alternative approach, GPT2 expression was knocked down in $CD4^+$ T cells with small interfering RNA (siRNA), which reduced mRNA abundance by 50% (Figure S3D). Similar to BCLA, GPT2 knockdown did not affect baseline frequencies of cytokine-expressing cells but it did again correct succinate-mediated inhibition of IFN-γ and TNF-α expression (Figure 3S). In a complementary approach, $CD4^+$ and $CD8^+$ T cells were activated in the presence of succinate together with excess pyruvate or dichloroacetate (DCA), which increases pyruvate mitochondrial oxidation by inhibiting pyruvate dehydrogenase kinase. Both of these approaches again rescued IFN-γ and TNF-α expression, confirming that impairing pyruvate flux through the TCA cycle does indeed play a role in the suppressive effects of succinate on T cells (Figures 3T, 3U, S3E, and S3F). Importantly, GC-MS analysis of [^{13}C]glucose-labeled $CD4^+$ T cells confirmed that BCLA suppressed ^{13}C incorporation into alanine in control and succinate-treated cells and that both BCLA and DCA increased glucose carbon labeling of succinate, fumarate, malate, aspartate, and glutamate (Figures S3G and S3H).

Having established that succinate exposure impairs $CD4^+$ and $CD8^+$ T cell function, we then investigated the relevance of this in PC and PG by combining *in vitro* models of the tumor microenvironment with interrogation of patient samples. To recreate the tumor micronenvironment *in vitro*, murine $CD4^+$ T cells were cultured in conditioned medium from wild-type (WT) or SDHB-deficient immortalized mouse chromaffin cells (imCC, clone 6 [CL6] and clone 8 [CL8]) (Klučková et al., 2020). These experiments identified suppression of IFN-γ and to a lesser extent TNF-α in T cells cultured in conditioned medium from SDHB-deficient CL6 and CL8 imCC compared with WT (Figure 4A), thus recapitulating the effects of exogenous succinate (Figure 1). Next, to investigate whether succinate impacts anti-tumor immunity in patients, we assessed RNA-sequencing data generated by Fishbein et al. (2017) from a subset of patients in their study (46 patients, 27% of their cohort) who had germline mutations in genes including SDHB (9%), RET (6%), VHL (4%), NF1 (3%) SDHD, MAX, EGLN1 (PHD2), and TMEM12 (all <2%). We did not assess data from patients in their cohort with somatic mutations, where no SDH mutations were present. Analysis of SDHB and SDHD transcript levels confirmed expected decreases in SDHB/SDHD expression in the presence of germline mutations in these genes (Figures S4A and S4B). Next, to assess the abundance of immune cell populations in an unbiased manner, we applied the CIBERSORTx approach (Chen et al., 2018). This is an established machine-learning deconvolution algorithm allowing resolution and quantification of closely related cell subsets within a gene expression mixture based on cell-type-specific gene signatures. Analysis of the 46 samples using

Figure 3. Succinate inhibits T cell effector function through inhibition of succinyl-CoA synthetase activity and subversion of mitochondrial glucose oxidation

(A and B) (A) $CD4^+$ and (B) $CD8^+$ T cells were activated for 72 h in the presence of succinate at indicated concentrations and assessed for mitochondrial membrane potential using MitoSpy Orange (MSO) (n = 6 independent donors).

(C–J) $CD4^+$ T cells activated as in (A) were assessed by extracellular flux for rates of mitochondrial oxygen consumption (OCR) and extracellular acidification (ECAR). (E) ATP-coupled OCR, (F) basal glycolysis, (G) ATP-coupled OCR/basal glycolysis, and (H–J) mitochondrial (mito), glycolytic (glyco), and total ATP synthesis rates were calculated. n = 4 independent donors.

(K) $CD4^+$ T cells activated as in (A) were washed and incubated with fully ^{13}C-labeled glucose for 6 h and assessed for MID of indicated metabolites by GC-MS (n = 6 independent donors).

(L–O) (L and M) Succinyl-CoA synthetase (SCS) and (N and O) fumarase activity were measured in $CD4^+$ T cells activated as in (A) (L and N, example traces; M and O, summary data for n = 7 and n = 5 independent donors, respectively).

(P and Q) $CD4^+$ T cells, activated in the presence of 10 mM sodium metavanadate (SCSi) were assessed for (P) SCS activity and (Q) IFN-γ secretion by ELISA (n = 3–4 independent donors, respectively).

(R–U) $CD4^+$ T cells were activated for 72 h in the presence of succinate and/or (R) 240 μM β-chloro-L-alanine (BCLA), (S) scrambled or GPT2-targeting siRNA, (T) pyruvate (10 mM), or (U) dichloroacetate (DCA, 10 mM) and assessed for IFN-γ and TNF-α expression by flow cytometry (n = 5–6 independent donors).

Bars represent mean data. Error bars (C and D) represent SEM. *$p < 0.05$, **$p < 0.01$.

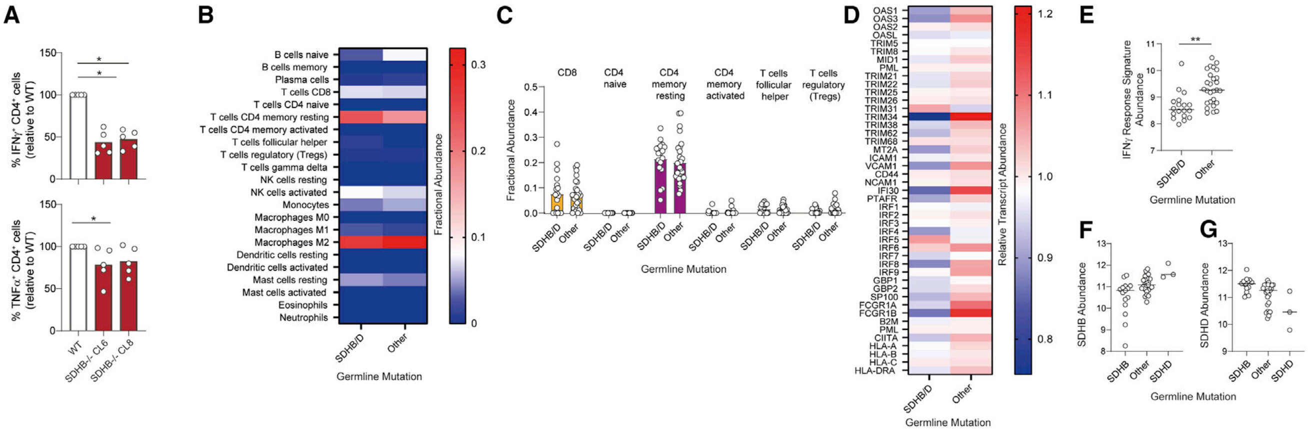

Figure 4. Tumor-derived succinate inhibits CD4+ T cell function, and pheochromocytomas and paragangliomas with loss-of-function SDH mutations demonstrate reduced expression of IFN-γ-induced genes

(A) Murine CD4+ T cells were activated for 48 h in conditioned medium from wild-type (WT) and SDHB-deficient (SDHB$^{-/-}$ CL6 and CL8) immortalized mouse chromaffin cells and assessed for IFN-γ and TNF-α expression by flow cytometry (n = 5 biological replicates from two independent experiments).

(B and C) CIBERSORTx analysis of fractional abundance of indicated immune cell subsets in tumor samples of pheochromocytoma and paraganglioma with germline mutations in SDHB/D (n = 18 patient samples) or other genes (n = 27 patient samples).

(D) Abundance of defined IFN-γ response transcripts within the same dataset expressed relative to the mean abundance of each transcript across all samples.

(E) Geometric mean of $\log_2$ normalized counts for the IFN-γ response signature transcripts in these samples.

(F and G) Log_2 normalized counts for SDHB (F) and SDHD (G) in samples with indicated germline mutations. Lines and bars represent mean data. *$p < 0.05$, **$p < 0.01$.

this approach identified no clear changes in immune cell subset abundance in SDHB/SDHD-deficient tumors compared with those without SDH mutations (Figure 4B) or indeed between any of the sample groups (Figure S4C). Interrogating T cell phenotypes in more detail also identified no clear changes between SDHB/SDHD-deficient tumors and those without SDH mutations (Figure 4D). This is perhaps not surprising, since our experiments revealed no effect of succinate on either T cell survival or proliferation. Indeed, since the most substantial effect of succinate was suppression of T cell-derived IFN-γ, we reasoned that this may result in altered transcription of IFN-γ response genes in tumor cells and tumor-infiltrating/resident cells. To assess for this, we constructed an IFN-γ response signature of 45 genes induced by IFN-γ signaling (for details see Table S2). Initial screening revealed that these genes were consistently downregulated in SDHB/SDHD-deficient tumors (Figures 4C and S4D). To quantify this effect across the whole gene signature, we generated a metric based on the geometric mean of the $\log_2$ normalized counts, which confirmed a substantial and highly significant decrease in SDHB/SDHD mutant tumors (Figure 4E). The extent to which the IFN-γ response signature was downregulated in SDHB/SDHD-deficient tumors was greater than either SDHD or SDHB, respectively (Figures 4F and 4G), similar in SDHB- and SDHD-deficient tumors (Figure S4E), and generally higher in all other groups, including the other pseudohypoxic group with VHL mutations (Figure S4F). Therefore, SDHB/SDHD mutant tumors demonstrate clearly decreased IFN-γ activity, consistent with local suppression of T cell IFN-γ expression by succinate. Transcripts for IFNG itself were scarce in all samples and often undetectable, preventing comparison of their abundance between tumor types; however, transcripts for IFNGR1 and IFNGR2 were readily detectable and similar across all tumor types (Figures S4G and S4H) indicating that decreased cytokine, rather than sensitivity to it, explains the suppressed IFN-γ response in SDHB/SDHD mutant tumors.

DISCUSSION

The TCA cycle intermediate succinate accumulates in tumors with loss-of-function mutations in SDH. These account for 10%–15% of inherited mutations in PC and PG, but SDH mutations are also present in 5% of gastrointestinal stromal tumors (Indio et al., 2021) and are reported in ovarian, renal, and thyroid cancer (Zhao et al., 2017). Additionally, elevated succinate levels are described in the absence of SDH mutations, including in lung, stomach, and colorectal cancers (Hirayama et al., 2009; Wu et al., 2019). Therefore, succinate-mediated inhibition of anti-tumor immunity may have broad implications across different types of malignancy. In a murine model of lung cancer, succinate accumulation was linked to the polarization of infiltrating macrophages toward a tumor-permissive phenotype (Wu et al., 2019). Indeed, potent effects of succinate on the innate immune system are well described, resulting from both SUCNR1-dependent and intracellular activity (Harber et al., 2020; Mills et al., 2016; Rubic et al., 2008; Tannahill et al., 2013).

In this study, we assessed the function of human CD4+ and CD8+ T cells activated under increasing succinate concentrations. We found that T cell viability, activation, and proliferation were not affected, but that degranulation, IFN-γ expression, and TNF-α expression were significantly inhibited. Consistent with this, CD4+ T cell cytokine expression was also impaired in the presence of conditioned medium from SDHB-deficient chromaffin cells, and interrogation of RNA-sequencing data from patient samples of PC and PG identified profound suppression of

IFN-γ-induced genes in tumors harboring loss-of-function mutations in SDH. These data support a role for succinate modulating anti-tumor immunity and are in agreement with recent data on gastrointestinal stromal tumors, where a similar IFN-γ-inducible gene signature was also lower in the context of SDH deficiency (Indio et al., 2021). To what extent this is caused by effects of succinate on T cells versus other IFN-γ-secreting immune cells cannot be established from these data, but the CIBERSORTx approach identified that the most abundant immune cell populations present in PC and PG are $CD4^+$ T cells and M2 macrophages. Since macrophage function is also highly sensitive to succinate, it would be pertinent to further interrogate implications of this in SDH-mutant disease.

In our experiments, inhibition of T cell function was partly SUCNR1 dependent and was also associated with significant succinate uptake by T cells. These results indicate that activated $CD4^+$ T cells readily assimilate environmental succinate and imply that targeting the relevant transporters may augment T cell function in succinate-rich environments. Here, we identify that $CD4^+$ T cell succinate uptake is partly MCT1 dependent, consistent with a recently identified role in mediating efflux of protonated succinate from muscle cells (Reddy et al., 2020), but not mediated by MCT4. The sodium-dependent dicarboxylate transporter SLC13A3 and citrate transporter SLC13A5 are also described to transport succinate in macrophages (Peruzzotti-Jametti et al., 2018). However, we find no detectable transcripts for these in resting or activated human $CD4^+$ and $CD8^+$ T cells.

Despite substantially increased intracellular succinate levels, we did not observe increased HIF-1α abundance or transcriptional activity in succinate-exposed T cells as is reported in macrophages (Tannahill et al., 2013). However, we did observe inhibited SCS activity, impaired glucose flux through the TCA cycle, and ATP-coupled oxygen consumption. It is now well established that mitochondrial glucose oxidation critically supports the effector functionality of activated T cells (Bantug et al., 2018a) through heightened generation of mitochondrial ATP and ROS as well as TCA cycle intermediates that alter chromatin accessibility and transcriptional status (Peng et al., 2016; Sena et al., 2013). Consistent with this, we observed that the inhibition of T cell cytokine secretion upon succinate exposure was associated with impaired glucose oxidation, decreased mitochondrial membrane potential, and suppressed mitochondrial and overall ATP synthesis. Moreover, restoration of TCA cycle flux reversed succinate-mediated suppression of T cell function, indicating that this is a key mechanism involved. Notably, a recent study identified decreased expression and activity of SCS in T cells from patients with rheumatoid arthritis. As in our experiments, this was also associated with decreased mitochondrial activity and membrane potential (Wu et al., 2020) but, alongside this, increased abundance of upstream citrate and its derivative acetyl-CoA were also linked to increased acetylation of tubulin and enhanced T cell motility, which we did not assess herein.

In summary, we report that T cells readily assimilate exogenous succinate when present at tumor-associated concentrations in their environment. This leads to inhibition of glucose flux through the TCA cycle, which, alongside SUCNR1 signaling, causes impaired T cell effector functionality. Consistently, SDH-deficient tumors demonstrate decreased expression of IFN-γ-inducible genes, which may contribute to their increased malignant potential. Targeting or prevention of succinate-induced metabolic T cell dysfunction may therefore represent promising novel approaches in tumors characterized by succinate accumulation.

Limitations of the study

Key functional and mechanistic observations regarding effects of succinate on T cells have been demonstrated here largely using human primary *in vitro* systems. *In vivo* validation of these findings will be important; however, it is reliant upon the further development of accurate experimental models of this disease. Conclusions about the relationship between SDH deficiency and T cell function in patients with PC or PG are based on *in silico* analysis of tumor RNA-sequencing data, and it would also be important to validate these data by analysis of T cells within tumor sections.

STAR★METHODS

Detailed methods are provided in the online version of this paper and include the following:

- KEY RESOURCES TABLE
- RESOURCE AVAILABILITY
  - Lead contact
  - Materials availability
  - Data and code availability
- EXPERIMENTAL MODEL AND SUBJECT DETAILS
  - Animals
  - Human studies
  - Cell lines
- METHOD DETAILS
  - $CD4^+$ and $CD8^+$ T cell isolation and culture
  - Flow cytometry analysis of protein expression, degranulation and proliferation
  - Multiplex cytokine analysis
  - IFN-γ ELISA
  - Assessment of mitochondrial membrane potential ($\Delta\Psi m$)
  - Extracellular flux analysis (Seahorse)
  - Assessment of reactive oxygen species (ROS)
  - Quantification of mRNA
  - Western blotting analysis
  - Stable isotope based metabolic tracing
  - mRNA silencing
  - Succinyl-CoA synthetase and fumarase assays
  - Analysis of RNA-sequencing data
- QUANTIFICATION AND STATISTICAL ANALYSIS

SUPPLEMENTAL INFORMATION

Supplemental information can be found online at https://doi.org/10.1016/j.celrep.2022.111193.

ACKNOWLEDGMENTS

This work was funded by a Marie Sklowdowska-Curie Actions individual fellowship to S.D. (Tracer-T). S.D. is also supported by a Birmingham

Fellowship. N.G. was supported by a Wellcome Trust ISSF continuity grant. D.B. is supported by an MRC Career Development award MR/V009052/1. J.R. and D.T. are supported by a CRUK program grant C42109/A24757 and the Paradifference Foundation. Wild-type and SDHB-deficient (SDHB$^{-/-}$ CL6 and CL8) immortalized mouse chromaffin cells (imCC) were generously provided by Prof. Judith Favier (INSERM, UMR970, Paris-Cardiovascular Research Center, Paris). The SUCNR1 inhibitor 4C was generously provided by Prof. S Pluchino (University of Cambridge, UK). Stable isotope-based tracing analysis was conducted at the Metabolic Tracer Analysis core facility at the University of Birmingham.

AUTHOR CONTRIBUTIONS

S.D. designed and conceptualized the experiments, performed *in silico* analyses, and wrote the paper. N.G., H.M., E.L.B., J.H., and T.F.-W. conducted experiments. D.B. assisted with data analysis, including that of RNA-sequencing data. J.R. and D.A.T. performed, analyzed, and interpreted GC-MS stable isotope tracing experiments.

DECLARATION OF INTERESTS

The authors declare no competing interests.

Received: December 6, 2021
Revised: May 26, 2022
Accepted: July 20, 2022
Published: August 16, 2022

REFERENCES

Bantug, G.R., Fischer, M., Grählert, J., Balmer, M.L., Unterstab, G., Develioglu, L., Steiner, R., Zhang, L., Costa, A.S.H., Gubser, P.M., et al. (2018a). Mitochondria-endoplasmic reticulum contact sites function as immunometabolic hubs that orchestrate the rapid recall response of memory CD8(+) T cells. Immunity *48*, 542–555.e6. https://doi.org/10.1016/j.immuni.2018.02.012.

Bantug, G.R., Galluzzi, L., Kroemer, G., and Hess, C. (2018b). The spectrum of T cell metabolism in health and disease. Nat. Rev. Immunol. *18*, 19–34. https://doi.org/10.1038/nri.2017.99.

Bunse, L., Pusch, S., Bunse, T., Sahm, F., Sanghvi, K., Friedrich, M., Alansary, D., Sonner, J.K., Green, E., Deumelandt, K., et al. (2018). Suppression of antitumor T cell immunity by the oncometabolite (R)-2-hydroxyglutarate. Nat. Med. *24*, 1192–1203. https://doi.org/10.1038/s41591-018-0095-6.

Caielli, S., Veiga, D.T., Balasubramanian, P., Athale, S., Domic, B., Murat, E., Banchereau, R., Xu, Z., Chandra, M., Chung, C.H., et al. (2019). A CD4(+) T cell population expanded in lupus blood provides B cell help through interleukin-10 and succinate. Nat. Med. *25*, 75–81. https://doi.org/10.1038/s41591-018-0254-9.

Chen, B., Khodadoust, M.S., Liu, C.L., Newman, A.M., and Alizadeh, A.A. (2018). Profiling tumor infiltrating immune cells with CIBERSORT. Methods Mol. Biol. *1711*, 243–259. https://doi.org/10.1007/978-1-4939-7493-1_12.

Finlay, D.K., Rosenzweig, E., Sinclair, L.V., Feijoo-Carnero, C., Hukelmann, J.L., Rolf, J., Panteleyev, A.A., Okkenhaug, K., and Cantrell, D.A. (2012). PDK1 regulation of mTOR and hypoxia-inducible factor 1 integrate metabolism and migration of CD8+ T cells. J. Exp. Med. *209*, 2441–2453. https://doi.org/10.1084/jem.20112607.

Fishbein, L., Leshchiner, I., Walter, V., Danilova, L., Robertson, A.G., Johnson, A.R., Lichtenberg, T.M., Murray, B.A., Ghayee, H.K., Else, T., et al. (2017). Comprehensive molecular characterization of pheochromocytoma and paraganglioma. Cancer Cell *31*, 181–193. https://doi.org/10.1016/j.ccell.2017.01.001.

Harber, K.J., de Goede, K.E., Verberk, S.G.S., Meinster, E., de Vries, H.E., van Weeghel, M., de Winther, M.P.J., and Van den Bossche, J. (2020). Succinate is an inflammation-induced immunoregulatory metabolite in macrophages. Metabolites *10*, 372. https://doi.org/10.3390/metabo10090372.

Hirayama, A., Kami, K., Sugimoto, M., Sugawara, M., Toki, N., Onozuka, H., Kinoshita, T., Saito, N., Ochiai, A., Tomita, M., et al. (2009). Quantitative metabolome profiling of colon and stomach cancer microenvironment by capillary electrophoresis time-of-Flight mass spectrometry. Cancer Res. *69*, 4918–4925.

Indio, V., Schipani, A., Nannini, M., Urbini, M., Rizzo, A., Leo, A.D., Altimari, A., Scioscio, V.D., Messelodi, D., Tarantino, G., et al. (2021). Gene expression landscape of SDH-deficient gastrointestinal stromal tumors. J. Clin. Med. *10*, 1057.

Keiran, N., Ceperuelo-Mallafré, V., Calvo, E., Hernández-Alvarez, M.I., Ejarque, M., Núñez-Roa, C., Horrillo, D., Maymó-Masip, E., Rodríguez, M.M., Fradera, R., et al. (2019). SUCNR1 controls an anti-inflammatory program in macrophages to regulate the metabolic response to obesity. Nat. Immunol. *20*, 581–592. https://doi.org/10.1038/s41590-019-0372-7.

Kľučková, K., Thakker, A., Vettore, L., Escribano-Gonzalez, C., Hindshaw, R.L., Tearle, J.L.E., Goncalves, J., Kaul, B., Lavery, G.G., Favier, J., and Tennant, D.A. (2020). Succinate dehydrogenase deficiency in a chromaffin cell model retains metabolic fitness through the maintenance of mitochondrial NADH oxidoreductase function. FASEB J. *34*, 303–315. https://doi.org/10.1096/fj.201901456R.

Křivánek, J., and Nováková, L. (1991). A novel effect of vanadium ions: inhibition of succinyl-CoA synthetase. Gen. Physiol. Biophys. *10*, 71–82.

Lei, W., Ren, W., Ohmoto, M., Urban, J.F., Matsumoto, I., Margolskee, R.F., and Jiang, P. (2018). Activation of intestinal tuft cell-expressed Sucnr1 triggers type 2 immunity in the mouse small intestine. Proc. Natl. Acad. Sci. USA *115*, 5552–5557. https://doi.org/10.1073/pnas.1720758115.

Letouzé, E., Martinelli, C., Loriot, C., Burnichon, N., Abermil, N., Ottolenghi, C., Janin, M., Menara, M., Nguyen, A.T., Benit, P., et al. (2013). SDH mutations establish a hypermethylator phenotype in paraganglioma. Cancer Cell *23*, 739–752. https://doi.org/10.1016/j.ccr.2013.04.018.

Li, X., Wu, F., and Beard, D.A. (2013). Identification of the kinetic mechanism of succinyl-CoA synthetase. Biosci. Rep. *33*, 145–163. https://doi.org/10.1042/BSR20120069.

Love, M.I., Huber, W., and Anders, S. (2014). Moderated estimation of fold change and dispersion for RNA-seq data with DESeq2. Genome Biol. *15*, 550. https://doi.org/10.1186/s13059-014-0550-8.

Matlac, D.M., Hadrava Vanova, K., Bechmann, N., Richter, S., Folberth, J., Ghayee, H.K., Ge, G.-B., Abunimer, L., Wesley, R., Aherrahrou, R., et al. (2021). Succinate mediates tumorigenic effects via succinate receptor 1: potential for new targeted treatment strategies in succinate dehydrogenase deficient paragangliomas. Front. Endocrinol. *12*, 589451. https://doi.org/10.3389/fendo.2021.589451.

Mills, E.L., Kelly, B., Logan, A., Costa, A.S.H., Varma, M., Bryant, C.E., Tourlomousis, P., Däbritz, J.H.M., Gottlieb, E., Latorre, I., et al. (2016). Succinate dehydrogenase supports metabolic repurposing of mitochondria to drive inflammatory macrophages. Cell *167*, 457–470.e13. https://doi.org/10.1016/j.cell.2016.08.064.

Nadjsombati, M., McGinty, J.W., Lyons-Cohen, M.R., Jaffe, J.B., DiPeso, L., Schneider, C., Miller, C.N., Pollack, J.L., Nagana Gowda, G.A., Fontana, M.F., et al. (2018). Detection of Succinate by Intestinal Tuft Cells Triggers a Type 2 Innate Immune Circuit. Immunity *49*, 33–41.e7. https://doi.org/10.1016/j.immuni.2018.06.016.

Peng, M., Yin, N., Chhangawala, S., Xu, K., Leslie, C.S., and Li, M.O. (2016). Aerobic glycolysis promotes T helper 1 cell differentiation through an epigenetic mechanism. Science *354*, 481–484. https://doi.org/10.1126/science.aaf6284.

Peruzzotti-Jametti, L., Bernstock, J.D., Vicario, N., Costa, A.S.H., Kwok, C.K., Leonardi, T., Booty, L.M., Bicci, I., Balzarotti, B., Volpe, G., et al. (2018). Macrophage-derived extracellular succinate licenses neural stem cells to suppress chronic neuroinflammation. Cell Stem Cell *22*, 355–368.e13. https://doi.org/10.1016/j.stem.2018.01.020.

Quinn, W.J., Jiao, J., TeSlaa, T., Stadanlick, J., Wang, Z., Wang, L., Akimova, T., Angelin, A., Schäfer, P.M., Cully, M.D., et al. (2020). Lactate limits T cell

proliferation via the NAD(H) redox state. Cell Rep. *33*, 108500. https://doi.org/10.1016/j.celrep.2020.108500.

Ratter, J.M., Rooijackers, H.M.M., Hooiveld, G.J., Hijmans, A.G.M., de Galan, B.E., Tack, C.J., and Stienstra, R. (2018). In vitro and in vivo effects of lactate on metabolism and cytokine production of human primary PBMCs and monocytes. Front. Immunol. *9*, 2564. https://doi.org/10.3389/fimmu.2018.02564.

Reddy, A., Bozi, L.H.M., Yaghi, O.K., Mills, E.L., Xiao, H., Nicholson, H.E., Paschini, M., Paulo, J.A., Garrity, R., Laznik-Bogoslavski, D., et al. (2020). pH-gated succinate secretion regulates muscle remodeling in response to exercise. Cell *183*, 62–75.e17. https://doi.org/10.1016/j.cell.2020.08.039.

Richter, S., Peitzsch, M., Rapizzi, E., Lenders, J.W., Qin, N., de Cubas, A.A., Schiavi, F., Rao, J.U., Beuschlein, F., Quinkler, M., et al. (2014). Krebs cycle metabolite profiling for identification and stratification of pheochromocytomas/paragangliomas due to succinate dehydrogenase deficiency. J. Clin. Endocrinol. Metab. *99*, 3903–3911. https://doi.org/10.1210/jc.2014-2151.

Rubic, T., Lametschwandtner, G., Jost, S., Hinteregger, S., Kund, J., Carballido-Perrig, N., Schwärzler, C., Junt, T., Voshol, H., Meingassner, J.G., et al. (2008). Triggering the succinate receptor GPR91 on dendritic cells enhances immunity. Nat. Immunol. *9*, 1261–1269. https://doi.org/10.1038/ni.1657.

Saraiva, A.L., Veras, F.P., Peres, R.S., Talbot, J., Lima, K.A., Luiz, J.P., Carballido, J.M., Cunha, T.M., Cunha, F.Q., Ryffel, B., and Alves-Filho, J.C. (2018). Succinate receptor deficiency attenuates arthritis by reducing dendritic cell traffic and expansion of Th17 cells in the lymph nodes. FASEB J. *32*, 6550–6558. https://doi.org/10.1096/fj.201800285.

Sciacovelli, M., and Frezza, C. (2016). Oncometabolites: unconventional triggers of oncogenic signalling cascades. Free Radic. Biol. Med. *100*, 175–181. https://doi.org/10.1016/j.freeradbiomed.2016.04.025.

Selak, M.A., Armour, S.M., MacKenzie, E.D., Boulahbel, H., Watson, D.G., Mansfield, K.D., Pan, Y., Simon, M.C., Thompson, C.B., and Gottlieb, E. (2005). Succinate links TCA cycle dysfunction to oncogenesis by inhibiting HIF-alpha prolyl hydroxylase. Cancer Cell *7*, 77–85. https://doi.org/10.1016/j.ccr.2004.11.022.

Sena, L.A., Li, S., Jairaman, A., Prakriya, M., Ezponda, T., Hildeman, D.A., Wang, C.R., Schumacker, P.T., Licht, J.D., Perlman, H., et al. (2013). Mitochondria are required for antigen-specific T cell activation through reactive oxygen species signaling. Immunity *38*, 225–236. https://doi.org/10.1016/j.immuni.2012.10.020.

Tannahill, G.M., Curtis, A.M., Adamik, J., Palsson-McDermott, E.M., McGettrick, A.F., Goel, G., Frezza, C., Bernard, N.J., Kelly, B., Foley, N.H., et al. (2013). Succinate is an inflammatory signal that induces IL-1beta through HIF-1alpha. Nature *496*, 238–242. https://doi.org/10.1038/nature11986.

Thiel, M., Caldwell, C.C., Kreth, S., Kuboki, S., Chen, P., Smith, P., Ohta, A., Lentsch, A.B., Lukashev, D., and Sitkovsky, M.V. (2007). Targeted deletion of HIF-1alpha gene in T cells prevents their inhibition in hypoxic inflamed tissues and improves septic mice survival. PLoS One *2*, e853. https://doi.org/10.1371/journal.pone.0000853.

Wu, B., Qiu, J., Zhao, T.V., Wang, Y., Maeda, T., Goronzy, I.N., Akiyama, M., Ohtsuki, S., Jin, K., Tian, L., et al. (2020). Succinyl-CoA ligase deficiency in pro-inflammatory and tissue-invasive T cells. Cell Metab. *32*, 967–980.e5. https://doi.org/10.1016/j.cmet.2020.10.025.

Wu, J.Y., Huang, T.W., Hsieh, Y.T., Wang, Y.F., Yen, C.C., Lee, G.L., Yeh, C.C., Peng, Y.J., Kuo, Y.Y., Wen, H.T., et al. (2020). Cancer-derived succinate promotes macrophage polarization and cancer metastasis via succinate receptor. Mol. Cell *77*, 213–227.e5. https://doi.org/10.1016/j.molcel.2019.10.023.

Zhao, T., Mu, X., and You, Q. (2017). Succinate: an initiator in tumorigenesis and progression. Oncotarget *8*, 53819–53828. https://doi.org/10.18632/oncotarget.17734.

STAR★METHODS

KEY RESOURCES TABLE

REAGENT or RESOURCE	SOURCE	IDENTIFIER
**Antibodies**		
anti-CD69-APC	Biolegend	Clone FN50, Cat# 310910; RRID:AB_314845
anti-CD25-BV605	Biolegend	Clone BC96, Cat# 302632; RRID:AB_11218989
anti-CD107a-FITC	BD Pharmingen	Clone H4A3, Cat# 560949; RRID:AB_2033934
anti-IFN-γ-FITC	Biolegend	Clone B27, Cat# 506504; RRID:AB_315437
anti-TNF-α-PE	Biolegend	Clone Mab11, Cat# 502909; RRID:AB_315261
anti-IFN-γ capture (ELISA)	Bio-Rad	Clone AbD00676, Cat# HCA043; RRID:AB_906045
anti-IFN-γ biotinylated (ELISA)	Bio-Rad	Clone 2503 Cat# HCA044P; RRID:AB_10614712
anti-HIF-1α [H1alpha67] (WB)	Abcam	Cat# ab1; RRID:AB_296474
anti-SUCNR1 (WB)	Novus Biologicals	Cat# NBP1-00861; RRID:AB_1503315
anti-CD3 (mouse)	Biolegend	Clone 145-2C11 Cat# 100340; RRID:AB_11149115
anti-CD28 (mouse)	Biolegend	Clone 37.51 Cat# 102116; RRID:AB_11147170
anti-IFN-γ-FITC (mouse)	Biolegend	Clone XMG1.2, Cat# 505806; RRID:AB_315400
anti-TNF-α-PE (mouse)	Biolegend	Clone MP6-XT22, Cat# 506305; RRID:AB_315426
**Chemicals, peptides, and recombinant proteins**		
RPMI1640 Cell Culture Medium	Sigma Aldrich	Cat# F9665
Penicillin/Streptomycin	Thermo Fisher	Cat# 15140122
Recombinant human IL-2	PeproTech	Cat# 200-02
Immunocult Human CD3/CD28 T cell activator	Stemcell Technologies	Cat# 10991
Sodium Succinate	Sigma Aldrich	Cat# 14160
AZD-3965	Selleckchem	Cat# S7339
β-chloro-L-alanine	Sigma Aldrich	Cat# C9033
Sodium Pyruvate	Sigma Aldrich	Cat# P8574
Dichloroacetate	Sigma Aldrich	Cat# 347795
Sodium Metavanadate	Sigma Aldrich	Cat# 72060
Cell Activation Cocktail	Biolegend	Cat# 423304
Fixation/permeabilization solutions	eBioscience	Cat# 00-5523-00
CellTrace Violet	Thermo Fisher	Cat# C34557
Recombinant IFN-γ standard (ELISA)	Bio-Rad	Cat# PHP050
streptavidin-HRP	Sigma Aldrich	Cat# E2866
TMB substrate	Bio-Rad	Cat# BUF062B
MitoSpy Orange	Biolegend	Cat# 424804
Laemmli buffer	Sigma Aldrich	Cat# 38733
RIPA buffer	Thermo Fisher Scientific	Cat# 89900
Clarity Western ECL Substrate	Bio-Rad	Cat# 170-5061
U-$^{13}C_6$ glucose	CK Isotopes	Cat# CLM-1396-1
$^{13}C_4$ sodium succinate	Sigma Aldrich	Cat# 491985
Methoxyamine hydrochloride in pyridine	Thermo Fisher Scientific	Cat# 25104
Lipofectamine RNAiMAX	Thermo Fisher	Cat# 13778075
Fixable Viability Dye eFluor™ 780	Thermo Fisher Scientific	Cat# 65-0865-14
4C (SUCNR1 antagonist)	Provided by Prof S Pluchino	N/A

(*Continued on next page*)

Continued		
REAGENT or RESOURCE	SOURCE	IDENTIFIER
Syrosingopine	Sigma Aldrich	Cat# SML1908
Critical commercial assays		
Qiagen spin columns	Qiagen	Cat# 27104
GPT2 and control scrambled siRNA	Origene	Cat# SR313641
Succinyl-CoA synthetase Assay	Biovision	Cat# K597
Fumarase Assay	Biovision	Cat# K596
Legendplex human Th1/2 panel kit	Biolegend	Cat# 741029
Deposited data		
RNA-sequencing of pheochromocytoma / paraganglioma tumors	Fishbein et al. (2017)	https://tcga-data.nci.nih.gov/docs/publications/pcpg_2016
Experimental models: Cell lines		
Wild-type and SDHB-deficient (Sdhb−/− CL6 and CL8) immortalized mouse Chromaffin Cells	Provided by Prof Judith Favier (INSERM, UMR970, Paris-Cardiovascular Research Center, Paris)	N/A
Biological samples		
Human primary peripheral blood mononuclear cells and CD4+/CD8+ T cells	Fully anonymised leukocyte cones collected from NHS Blood and Transplant (NHSBT), Birmingham, UK or healthy volunteer blood donors.	N/A
Experimental models: Organisms/strains		
Mouse: C57BL/6	Charles River Laboratories	Strain Code: 027
Oligonucleotides		
GLUT3 FWD GCTGGGCATCGTTGTTGGA	This Paper	N/A
GLUT3 REV GCACTTTGTAGGATAGCAGGAAG	This Paper	N/A
GPT2 FWD GACCCCGACAACATCTACCTG	This Paper	N/A
GPT2 REV TCATCACACCTGTCCGTGACT	This Paper	N/A
BNIP3 FWD CAGGGCTCCTGGGTAGAACT	This Paper	N/A
BNIP3 REV CTACTCCGTCCAGACTCATGC	This Paper	N/A
Software and algorithms		
CIBERSORTx	https://cibersortx.stanford.edu/	LM22 (22 immune cell subtypes) signature matrix file used.

RESOURCE AVAILABILITY

Lead contact

Further information and requests for resource and reagents should be directed to and will be fulfilled by the Lead Contact Dr. Sarah Dimeloe (s.k.dimeloe@bham.ac.uk).

Materials availability

This study did not generate new unique reagents.

Data and code availability

- This paper analyzes existing, publicly available data. These accession numbers for the datasets are listed in the key resources table.
- This paper does not report original code.
- Any additional information required to reanalyze the data reported in this paper is available from the lead contact upon request.

CellPress
OPEN ACCESS

EXPERIMENTAL MODEL AND SUBJECT DETAILS

Animals

Splenic $CD4^+$ T cells were isolated from C57BL/6 mice between 6 and 10 weeks of age. Equal numbers of male and female mice were used. All animal experiments were approved by the local animal welfare and ethical review body. Animals were housed in specific pathogen-free conditions.

Human studies

Human peripheral blood mononuclear cells were isolated from fully anonymised leukocyte cones (information on age and sex not provided) collected from NHS Blood and Transplant (NHSBT), Birmingham, UK or healthy volunteer blood donors. All volunteers signed a consent form, and all studies were approved by the University of Birmingham STEM Ethics Committee (Ref. ERN 17_1743).

Cell lines

Wild-type and SDHB-deficient (SDHB−/− CL6 and CL8) immortalized mouse Chromaffin Cells (imCC) were kindly provided by Prof Judith Favier (INSERM, UMR970, Paris-Cardiovascular Research Center, Paris). These had been generated by transfecting Wild-Type imCC with in silico designed targeted gRNA, using the PrecisionX Cas9 SmartNuclease RNA System Kit (System Biosciences) and screened by SDH activity test, qRT-PCR, and Sanger sequencing. These cells were cultured in DMEM Glutamax (Gibco) with 10% foetal calf serum (Sigma Aldrich Cat# F9665) and 1 mM pyruvate (Sigma Aldrich, Cat# P2256). Supernatants were taken at confluence and stored at −20°C.

METHOD DETAILS

$CD4^+$ and $CD8^+$ T cell isolation and culture

$CD4^+$ and $CD8^+$ T cells were isolated from human peripheral blood by density-gradient centrifugation and positive selection using CD4 or CD8 microbeads (Miltenyi Cat# 130-045-101 and Cat# 130-045-201). Purity was typically >95%. Cells were (unless otherwise indicated) resuspended at 1 × 10^6/mL in RPMI1640 containing 10% fetal calf serum (Sigma Aldrich Cat# F9665), 50 U/mL penicillin and 50 mg/mL streptomycin (Thermo Fisher, Cat# 15140122) (RPMI/10%FCS), and 50 IU/mL rIL-2 (PeproTech, Cat# 200-02). Where indicated, T cells were stimulated with Immunocult Human CD3/CD28 T cell activator (Stemcell Technologies, Cat# 10991) according to the manufacturer's instructions. Additions to cell culture included sodium succinate 0.5-5 mM (Sigma Aldrich, Cat# 14160), AZD-3965 10 μM (Selleckchem, Cat# S7339), syrosingopine (10 μM, Sigma Aldrich Cat#SML1908) β-chloro-L-alanine (BCLA, 240 μM, Sigma Aldrich, Cat# C9033), sodium pyruvate 10 mM (Sigma Aldrich, Cat# P8574), dichloroacetate 10 mM (Sigma Aldrich, Cat# 347795) sodium metavanadate 10 mM (SCSi, Sigma Aldrich Cat# 72060) and 4C 5 μM (Eurofins Advinus – kindly provided by Prof S Pluchino, University of Cambridge).

Murine $CD4^+$ T cells were isolated from wild-type C57BL/6 spleens by positive selection using CD4 microbeads (Miltenyi, Cat# 130-104-454). These were cultured in conditioned medium from Wild-type and SDHB-deficient (Sdhb−/− CL6 and CL8) immortalized mouse Chromaffin Cells (imCC) and stimulated with anti-CD3 and anti-CD28 monoclonal antibodies (plate-bound, 1 and 5 μg/mL, Biolegend, anti-CD3 Clone 145-2C11 Cat# 100340; anti-CD28 Clone 37.51 Cat# 102116).

Flow cytometry analysis of protein expression, degranulation and proliferation

CD69 and CD25 cell surface protein expression was assessed by flow cytometry (FACS) with specific monoclonal antibodies (anti-CD69-APC, Biolegend, Clone FN50, Cat# 310910; anti-CD25-BV605, Biolegend, Clone BC96, Cat# 302632). Cells (0.2 × 10^6 per condition) were stained for 20 min at 4°C in FACS buffer (1xPBS, 1% FCS) prior to washing and analysis. Degranulation was assessed as externalisation of CD107a by incubation with CD107a-FITC (BD Pharmingen, Clone H4A3, Cat# 560949) during a 5 hour stimulation period (0.2 × 10^6 cells per condition). For assessment of IFN-γ and TNF-α expression by intracellular cytokine staining, cells (0.2 × 10^6) were treated for 4 hours with Cell Activation Cocktail (with Brefeldin A, Biolegend Cat# 423,304). The cells were then washed and fixed for 20 min at room temperature with fixation/permeabilization solution and washed with permeabilization buffer (both from eBioscience Cat# 00-5523-00) before being stained for 20 min with anti-IFN-γ and anti-TNF-α (anti-IFN-γ-FITC, Biolegend, Clone B27, Cat# 506504; anti-TNF-α-PE, Biolegend, Clone Mab11, Cat# 502909) and then undergoing further washing and analysis. T cell proliferation was assessed by the dilution of CellTrace Violet (CTV) dye (Thermo Fisher, Cat# C34557). Cells (0.2 × 10^6 per condition) were labelled with CTV prior to cell culture, according to the manufacturer's instructions and dye dilution was measured at 72 h by flow cytometry.

For assessment of IFN-γ and TNF-α expression by intracellular cytokine staining in murine T cells, cells (0.2 × 10^6) were treated, fixed and permeabilised as above before being stained for 20 min with anti-IFN-γ and anti-TNF-α (anti-IFN-γ-FITC, Biolegend, Clone XMG1.2, Cat# 505806; anti-TNF-α-PE, Biolegend, Clone MP6-XT22, Cat# 506305) and then undergoing further washing and analysis.

Multiplex cytokine analysis

Cell culture supernatants from 0.2 × 10^6 cells were harvested at 72 h and stored at −20°C. The concentration of IL-2, IL-6, IL-10, IL-4, IL-5 and IL-13 were measured using the Legendplex human Th1/2 panel kit (Biolegend, Cat# 741029) according to the

manufacturer's instructions and using a BD Fortessa flow cytometer. Analysis was performed with FlowJo 10.0.8 (Tree Star, USA). Cytokine concentrations were corrected for cell count using known input cell number and measured percentage viability assessed by flow cytometry (i.e. exclusion of Fixable Viability Dye eFluor 780, Thermo Fisher Scientific, Cat# 65-0865-14).

IFN-γ ELISA

Cell culture supernatants from 0.2×10^6 cells were harvested at 72 h and stored at −20°C. The concentration of IFN-γ was measured by ELISA, using anti-IFN-γ capture (Bio-Rad, Clone AbD00676, Cat# HCA043) and biotinylated detection (Bio-Rad, Clone 2503 Cat# HCA044P) antibodies, recombinant IFN-γ standard (Bio-Rad, Cat# PHP050), streptavidin-HRP (Sigma Aldrich, Cat# E2866) and TMB substrate (Bio-Rad, Cat# BUF062B). Cytokine concentrations were corrected for cell count using known input cell number and measured percentage viability assessed by flow cytometry (i.e. exclusion of Fixable Viability Dye eFluor 780, Thermo Fisher Scientific, Cat# 65-0865-14).

Assessment of mitochondrial membrane potential (ΔΨm)

Cells were stained with MitoSpy Orange (MSO, Biolegend Cat# 424804) to assess for differences in mitochondrial membrane potential (ΔΨm). Cells (0.2×10^6) were incubated in RPMI 1640/10% FCS with 25 nM MSO for 20 min at 37°C and 5% CO_2 before undergoing washing and analysis by flow cytometry.

Extracellular flux analysis (Seahorse)

For analysis of the oxygen consumption rate (OCR, pmol/min) and extracellular acidification rate (ECAR, mpH/min), the Seahorse XFe96 metabolic extracellular flux analyzer was used (Agilent). $CD4^+$ T cells, previously activated with 0 or 5 mM succinate were resuspended in serum-free, unbuffered RPMI 1640 (Agilent) and were plated onto Seahorse cell plates (2.5×10^5 cells per well) coated with poly-d-lysine (Invitrogen) to enhance T cell attachment. Perturbation profiling of the use of metabolic pathways was done by the addition of oligomycin (1 μM), Bam-15 (3 μM), and rotenone/antimycin A (both 2 μM; all are given as final concentrations, all from Sigma-Aldrich). ATP-coupled OCR was calculated as the mean of the 3 measurements before oligomycin injection minus the mean of the 3 measurements after oligomycin injection. Basal glycolysis was calculated as the mean of the 3 measurements after glucose injection minus the mean of the 3 measurements before glucose injection. Rates of mitochondrial, glycolytic and total ATP synthesis were calculated from measured OCR and ECAR data using Agilent Wave Software.

Assessment of reactive oxygen species (ROS)

Cells were stained with 2',7'-Dichlorofluorescin Diacetate (DCFDA, Sigma Cat# 287810) to assess for differences in ROS. Cells (0.2×10^6) were incubated in RPMI 1640/10% FCS with 20 μM for 20 min at 37°C and 5% CO_2 before undergoing washing and analysis by flow cytometry.

Quantification of mRNA

The relative abundances of mRNAs of interest were quantified by real-time RT-PCR (qPCR). The mRNA was extracted with Qiagen spin columns (Qiagen, Cat# 27104) from 4×10^6 cells and cDNA was transcribed with the Promega reverse transcription reagents (PCR mix, Cat# U110A; Oligo dt primer, Cat# C110A; Rev transcriptase, Cat# M170A; RNasin, Cat# N261A) according to the manufacturer's instructions. SYBR green primers were used for qPCR analysis. See Table S1 for further details.

Western blotting analysis

Cell lysates from 4×10^6 cells per condition were prepared in laemmli buffer (for HIF-1α, Sigma Aldrich, Cat# 38733) or RIPA buffer (for SUCNR1, Thermo Fisher Scientific, Cat# 89900) and protein concentrations were determined with a BCA protein assay kit (for samples in RIPA only, Thermo Fisher Scientific, Cat# 23225). Whole-cell lysates were resolved by 10% SDS-PAGE and were transferred onto nitrocellulose membranes. The membranes were then incubated with anti-HIF-1α primary antibody (1/200, Abcam Cat# GR3364074-1) or anti-SUCNR1 primary antibody (1/500, Novus Biologicals Cat# NBP1-00861). The membranes were then stained with the appropriate secondary antibodies. The HRP-ECL system (Bio-Rad Clarity Western ECL Substrate Cat# 170–5061) was used for band detection.

Stable isotope based metabolic tracing

Tracing experiments were performed for the time indicated in the figure legend, in basic formulation RPMI supplemented with either 10 mM U-$^{13}C_6$ glucose (CK Isotopes, Cat# CLM-1396-1) or 5 mM U-$^{13}C_4$ sodium succinate (Sigma Aldrich Cat# 491,985). 4×10^6 cells per condition were labelled as indicated and were then washed with ice-cold 0.9% saline solution and were extracted in 1:1:1 pre-chilled methanol, HPLC-grade water (containing 1 μg/mL D6-glutaric acid) and chloroform. The extracts were shaken at 1400 rpm for 20 min at 4°C and centrifuged at 16,000 g for 5 min at 4°C. 0.3 mL of the upper aqueous phase was collected and evaporated under vacuum. Metabolite derivatization was performed using an Agilent autosampler. Dried polar metabolites were dissolved in 15 μL of 2% methoxyamine hydrochloride in pyridine (Thermo Fisher Scientific, Cat# 25104) at 55°C, followed by an equal volume of N-tert-Butyldimethylsilyl-N-methyltrifluoroacetamide with 1% tertbutyldimethylchlorosilane after 60 minutes, and incubation for a further 90 min at 55°C. GC-MS analysis was performed using an Agilent 6890GC equipped with a 30m DB-35 MS capillary column.

The GC was connected to an Agilent 5975C MS operating under electron impact ionization at 70 eV. The MS source was held at 230°C and the quadrupole at 150°C. The detector was operated in scan mode and 1 μL of derivatised sample was injected in splitless mode. Helium was used as a carrier gas at a flow rate of 1 mL/min. The GC oven temperature was held at 80°C for 6 min and increased to 325°C at a rate of 10°C/min for 4 min. The run time for each sample was 59 min. For determination of the mass isotopomer distributions (MIDs), spectra were corrected for natural isotope abundance. Data processing was performed using MATLAB.

mRNA silencing

siRNA targeting human GPT2 and control scrambled siRNA (Origene, Cat# SR313641) were delivered into primary human CD4+ T cells by transfection using Lipofectamine RNAiMAX (Thermo Fisher, Cat# 13778075) according to the manufacturer-provided protocol. 2.0×10^6 cells per condition were transfected for qPCR measurement of GPT2 mRNA abundance and 0.2×10^6 cells per condition for flow cytometric analysis of cytokine expression.

Succinyl-CoA synthetase and fumarase assays

Succinyl-CoA synthetase and Fumarase activity were measured in 5.0×10^6 cells, using assays (Biovision, Cat# K597 and Cat# K596 respectively) according to the manufacturer's instructions.

Analysis of RNA-sequencing data

HTSeq count data from PC and PG samples were accessed from the Genomic Data Commons Data Portal using the unique identifiers provided in (Fishbein et al., 2017). FPKM data were entered into the CIBERSORTx algorithm and the LM22 (22 immune cell subtypes) signature matrix file used to impute immune cell fractional abundance present. Log2 normalised counts were generated from raw HTSeq count data using the Bioconductor package DESeq2 (Love et al., 2014). For individual genes, the log2 normalised count data are reported and for the IFN-γ response signature (further details in Table S2) the geometric mean of the log2 normalised count data for each gene was calculated.

QUANTIFICATION AND STATISTICAL ANALYSIS

Data were analysed in FlowJo v10 (Tree Star Inc), Graphpad Prism 8 and 9 and R. Data with normal distribution were assessed by paired Student's two-sided t test. Multiple groups were compared by one- or two-way ANOVA and a Bonferroni post-test for multiple comparisons. Non-normally distributed data were compared using a Wilcoxon test. *p=<0.05, **p=<0.01, ***p=<0.001.

Cell Reports, Volume *40*

Supplemental information

Succinate uptake by T cells suppresses their effector function via inhibition of mitochondrial glucose oxidation

Nancy Gudgeon, Haydn Munford, Emma L. Bishop, James Hill, Taylor Fulton-Ward, David Bending, Jennie Roberts, Daniel A. Tennant, and Sarah Dimeloe

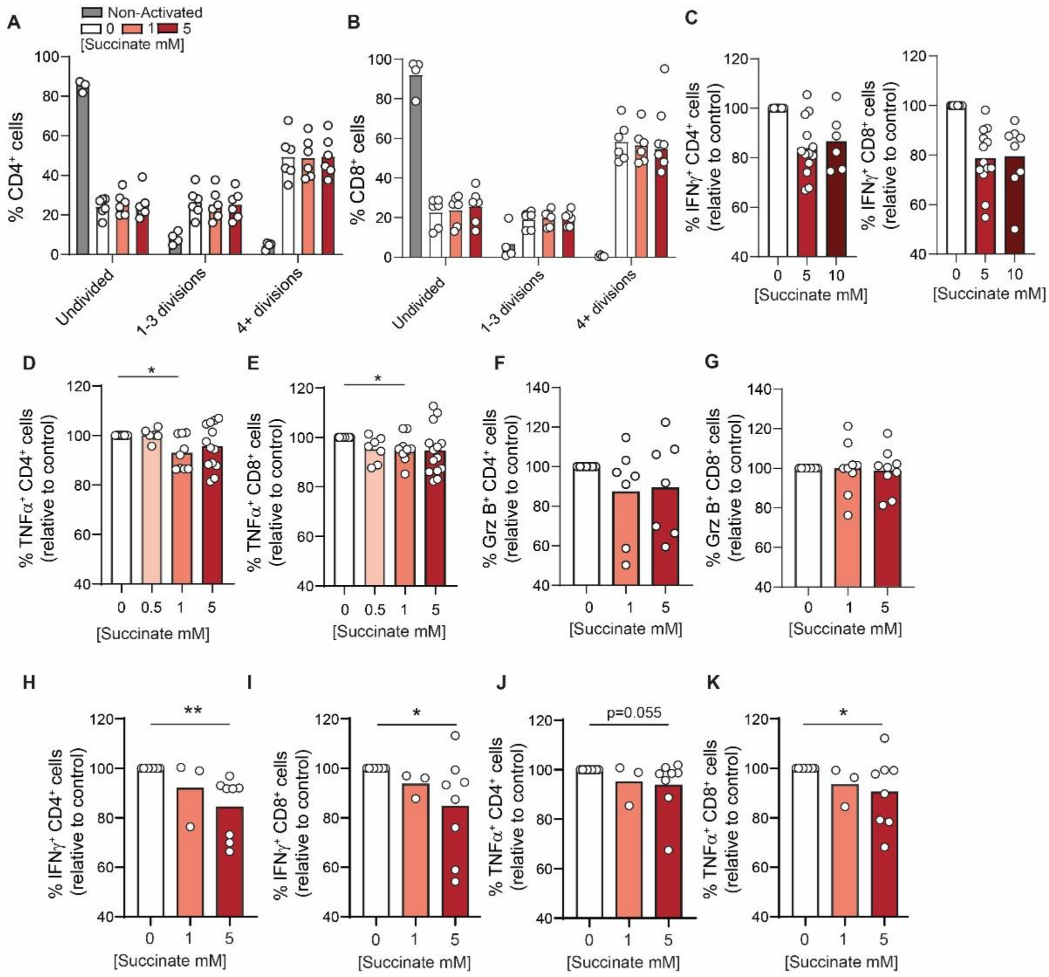

Supplementary Figure 1, related to Figure 1: Effects of succinate on CD4+ and CD8+ T cell proliferation and effector functions.

$CD4^+$ and $CD8^+$ T cells were activated for 72h *in vitro* in presence of exogenous succinate at indicated concentrations, and assessed for **(A-B)** proliferation (dilution of cell-trace violet) (n= 6 independent donors), **(C)** frequency of IFN-γ-expressing cells (n= 6 to 14 independent donors) **(D-E)** frequency of TNF-α-expressing cells (n= 6 to 14 independent donors) and **(F-G)** Granzyme B (GrzB) expressing cells (n= 6 to 9 independent donors) by flow cytometry. **(H-K)** T cells within total human PBMC were activated for 48h in presence of exogenous succinate at indicated concentrations and assessed for frequency of IFN-γ-expressing cells and TNF-α-expressing cells (n= 8 independent donors) by flow cytometry. Bars represent mean data. *p<05, **p<0.01

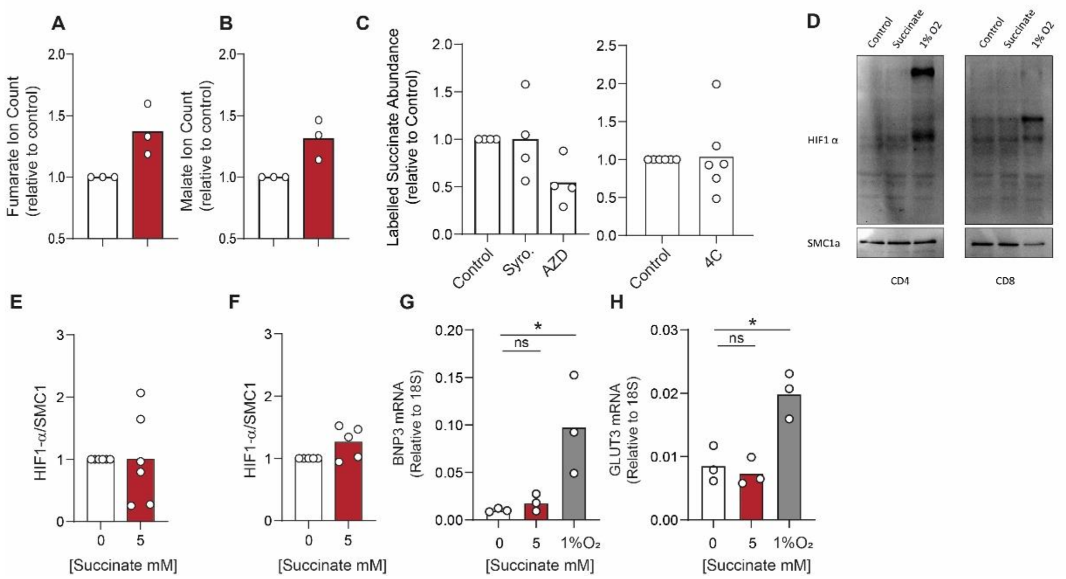

Supplementary Figure 2, related to Figure 2: Effect of succinate on intracellular TCA cycle intermediate abundance and HIF-1α activity in T cells

(A-B) CD4⁺ T cells were activated for 72h in vitro in presence of 5mM succinate as indicated and assessed for intracellular fumarate and malate abundance (expressed as ion count normalised to the internal standard, D-6-glutaric acid) by GC-MS (n=3 independent donors) **(C)** $CD4^+$ T cells were activated for 72h in presence of 5mM fully ^{13}C-labeled succinate in absence and presence of the MCT1/4 inhibitor syrosingopine (10µM), MCT1 inhibitor AZD-3965 (10µM) or SUCNR1 inhibitor 4C (5µM) and assessed for mass isotopomer distribution (MID) of the succinate by GC-MS (n=3 independent donors). (**D-F**) $CD4^+$and $CD8^+$ T cells were activated for 72h *in vitro* in presence of 5mM succinate and assessed for abundance of HIF-1α by western blot (n=5 independent donors). **(G-H)** $CD4^+$ T cells cultured as in (A) and additionally at 1% atmospheric O_2 were assessed for expression of **(G)** BNIP3 and **(H)** Glut3 by qPCR (n=3 independent donors). Bars represent mean data. *p<05, **p<0.01.

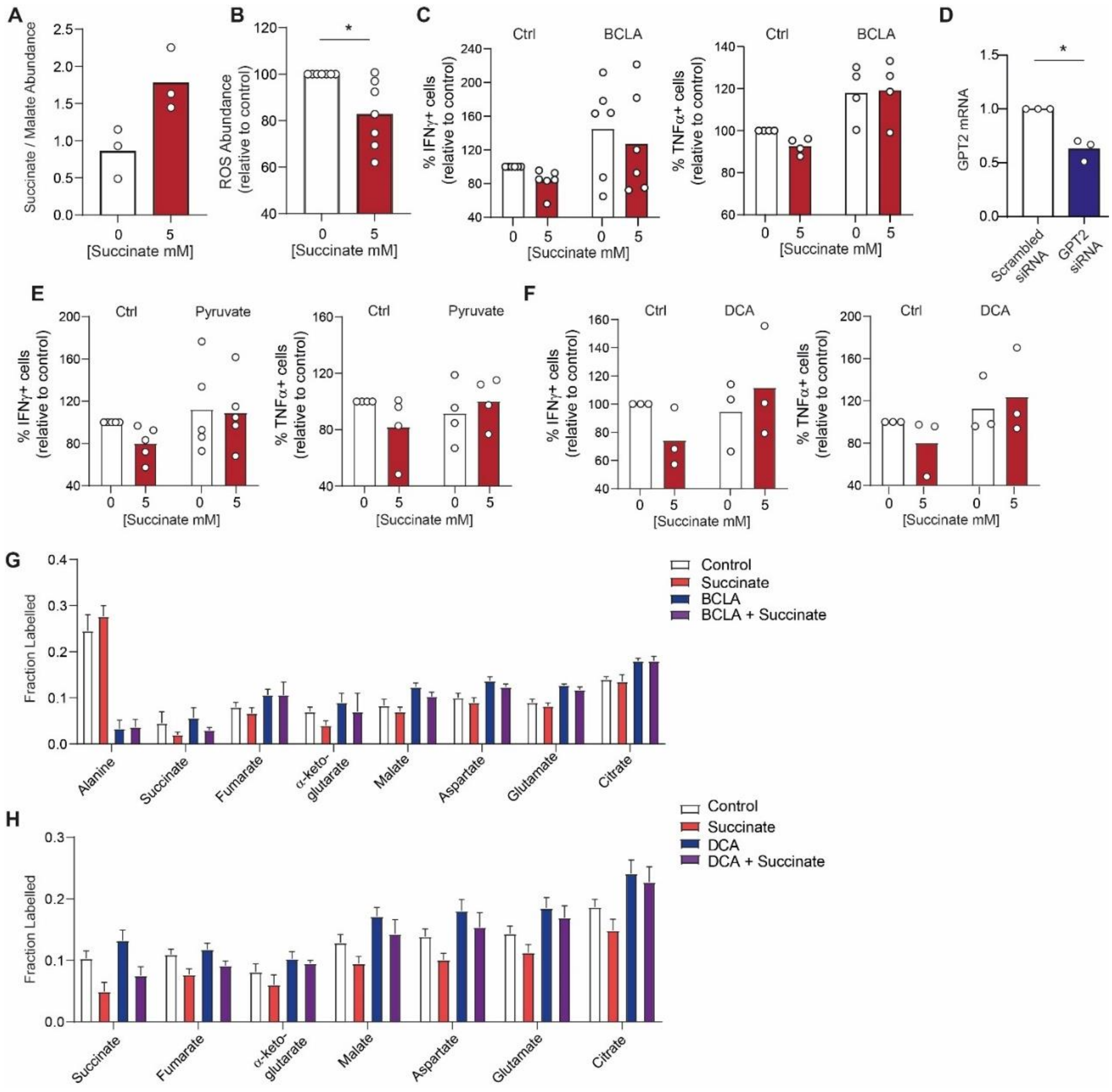

Supplementary Figure 3, related to Figure 3: Succinate impairs T cell mitochondrial activity; interventions to restore mitochondrial glucose oxidation restore T cell effector function

(A) CD4^{+} T cells were activated for 72h in vitro in presence of 5mM succinate as indicated and assessed for intracellular succinate and malate abundance (expressed as a ratio of the ion counts normalised to the internal standard, D-6-glutaric acid) by GC-MS (n=3 independent donors). (**B**) CD4+ T cells were activated as in (A) and assessed for total ROS abundance by flow cytometry following incubation with 2',7'–dichlorofluorescin diacetate (DCFDA) (n=7 independent donors) **(C)** CD8^{+} T cells were activated for 72h *in vitro* in presence of exogenous succinate and/or 240µM of the GPT2 inhibitor β-chloro-L-alanine (BCLA) and assessed for assessed for the frequency of IFN-γ- and TNF-α-expressing cells by flow cytometry (n=4 independent donors). **(D)** CD4^{+} T cells were activated for 72h in vitro in presence of exogenous succinate and/or scrambled or GPT2-targeting siRNA and assessed for GPT2 mRNA abundance by qPCR (n=3 independent donors) **(E-F)** CD8^{+} T cells were activated as in (D) in presence of succinate and/or pyruvate (10mM) or DCA (10mM) as indicated and assessed for the frequency of IFN-γ- and TNF-α-expressing cells by flow cytometry (n=4/3 independent donors for pyruvate/DCA respectively). Bars represent mean data. **(G-H)** CD4^{+} T cells were activated for 72h in vitro in presence of 5mM exogenous succinate and/or 240µM β-chloro-L-alanine (BCLA) or 10mM dichloroacetate (DCA) before incubation with fully ^{13}C-labelled glucose assessment of fractional labelling of the indicated metabolites by GC-MS (n=3 independent donors) Mean data +/-SEM are shown for G and H. *p<05, **p<0.01.

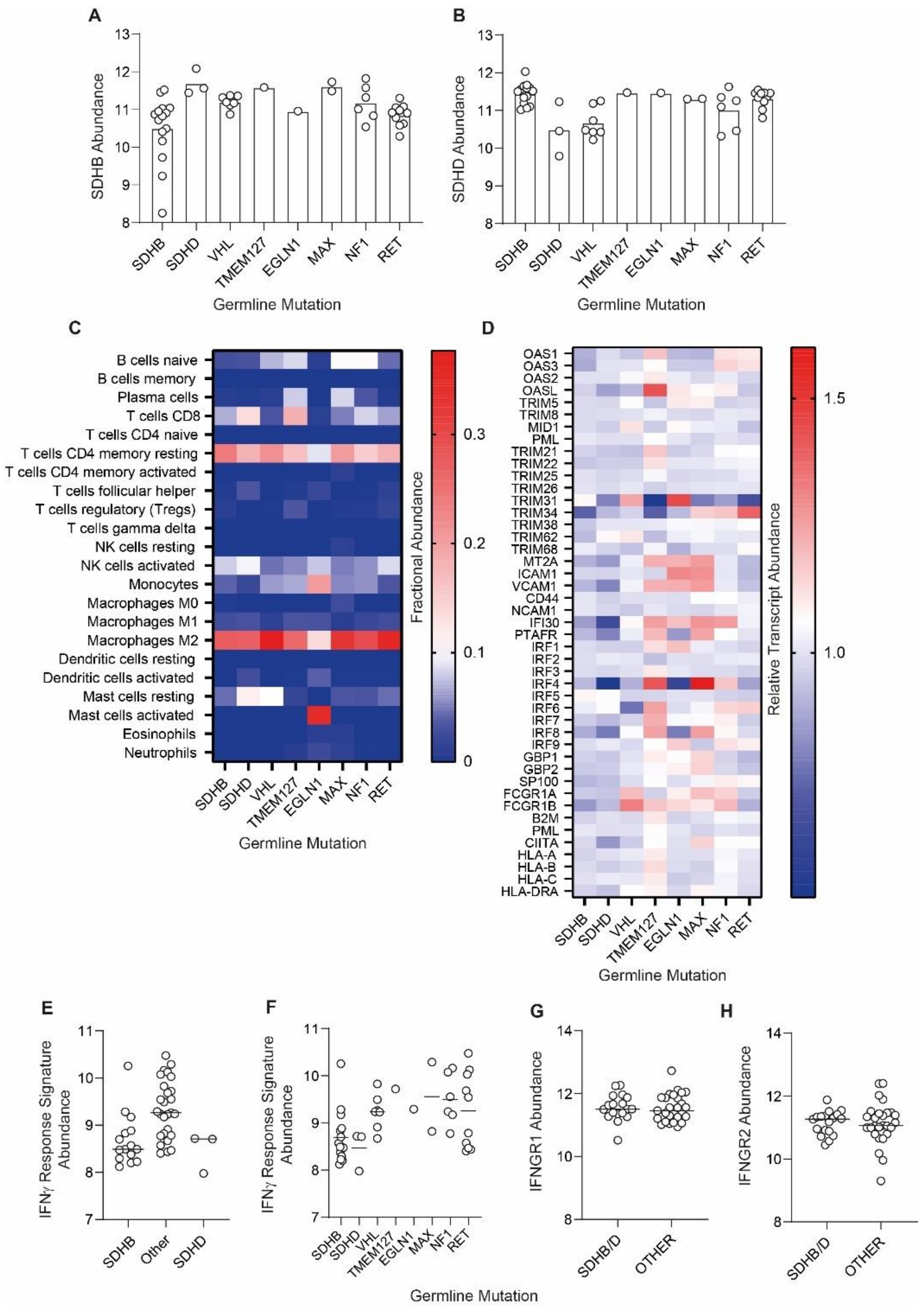

Supplementary Figure 4, related to Figure 4: Gene expression data and immune cell fractional abundance in pheochromocytoma and paraganglioma with germline mutations in SDHB, SDHD or other genes

(A-B) Log2normalised counts for SDHB **(A)** and SDHD **(B)** in tumour samples of pheochromocytoma and paraganglioma with the indicated germline mutations, using RNA-sequencing data generated by Fishbein et al[1]. **(C)** CIBERSORTx analysis of the fractional abundance of indicated immune cell subsets in these samples. **(D)** Abundance of defined IFN-γ response signature transcripts within the same dataset, expressed relative to the mean abundance of each transcript across all samples. **(E-F)** Geometric mean of Log2normalised counts for the IFN-γ response transcripts in these samples, comparing SDHB, SDHD and other germline mutations **(E)** or comparing all germline mutations separately **(F)**. **(G-H)** Log2normalised counts for IFNGR1 **(G)** and IFNGR2 **(H)** in samples with the indicated germline mutations Lines represent mean data.

Supplementary Table 1, related to STAR methods: Details of primers used for SYBRgreen qPCR analysis.

Gene	Forward	Reverse
BNIP3	CAGGGCTCCTGGGTAGAACT	CTACTCCGTCCAGACTCATGC
GLUT3	GCTGGGCATCGTTGTTGGA	GCACTTTGTAGGATAGCAGGAAG
GPT2	GACCCCGACAACATCTACCTG	TCATCACACCTGTCCGTGACT

Supplementary Table 2, related to Figure 4 and supplementary Figure 4: Details of IFN-γ signature genes in Fig. 4/Supp. Fig. 4, constructed using Reactome pathway browser

**Gene**	**Protein**
OAS1	2'-5'-oligoadenylate synthetase 1
OAS3	2'-5'-oligoadenylate synthetase 3
OAS2	2'-5'-oligoadenylate synthetase 2
OASL	2'-5'-oligoadenylate synthase-like protein
TRIM5	Tripartite motif-containing protein 5
TRIM8	Tripartite motif-containing protein 8
MID	MID1 / Tripartite motif-containing protein 18
PML	PML Nuclear Body Scaffold/ Tripartite motif-containing protein 19
TRIM21	Tripartite motif-containing protein 21
TRIM22	Tripartite motif-containing protein 22
TRIM25	Tripartite motif-containing protein 23
TRIM26	Tripartite motif-containing protein 26
TRIM31	Tripartite motif-containing protein 31
TRIM34	Tripartite motif-containing protein 34
TRIM38	Tripartite motif-containing protein 38
TRIM62	Tripartite motif-containing protein 62
TRIM68	Tripartite motif-containing protein 68
MT2A	Metallothienin 2A
ICAM1	Intercellular Adhesion Molecule 1
VCAM1	Vascular Cell Adhesion Molecule 1
CD44	CD44 / HCAM (homing cell adhesion molecule)
NCAM1	Neural Cell Adhesion Molecule 1
IFI30	Gamma-interferon-inducible lysosomal thiol reductase
PTAFR	Platelet-activating factor receptor
IRF1	Interferon regulatory factor 1
IRF2	Interferon regulatory factor 2
IRF3	Interferon regulatory factor 3
IRF4	Interferon regulatory factor 4
IRF5	Interferon regulatory factor 5
IRF6	Interferon regulatory factor 6
IRF7	Interferon regulatory factor 7
IRF8	Interferon regulatory factor 8
IRF9	Interferon regulatory factor 9
GBP1	Guanylate-binding protein 1
GBP2	Guanylate-binding protein 2
SP100	Nuclear autoantigen Sp-100
FCGR1A	High affinity immunoglobulin gamma Fc receptor I
FCGR1B	High affinity immunoglobulin gamma Fc receptor IB
B2M	Beta 2 microglobulin
PML	Protein PML
CIITA	MHC class II transactivator
HLA-A	HLA-A
HLA-B	HLA-B
HLA-C	HLA-C
HLA-DRA	HLA-DR
